# Approximating inverse FEM matrices on non-uniform meshes with ${\mathcal{H}}$-matrices

**DOI:** 10.1007/s10092-021-00413-w

**Published:** 2021-06-30

**Authors:** Niklas Angleitner, Markus Faustmann, Jens Markus Melenk

**Affiliations:** Technische Universität Wien, Institute of Analysis and Scientific Computing (Inst. E 101), Wiedner Hauptstrasse 8-10, A–1040 Wien, Austria

**Keywords:** FEM, ${\mathcal{H}}$-matrices, Approximability, Non-uniform meshes, Primary: 65F50, Secondary: 65F30, 65N30

## Abstract

We consider the approximation of the inverse of the finite element stiffness matrix in the data sparse ${\mathcal{H}}$-matrix format. For a large class of shape regular but possibly non-uniform meshes including algebraically graded meshes, we prove that the inverse of the stiffness matrix can be approximated in the ${\mathcal{H}}$-matrix format at an exponential rate in the block rank. Since the storage complexity of the hierarchical matrix is logarithmic-linear and only grows linearly in the block-rank, we obtain an efficient approximation that can be used, e.g., as an approximate direct solver or preconditioner for iterative solvers.

## Introduction

Discretizations of elliptic partial differential equations on a domain $\varOmega \subseteq \mathbb {R}^d$ using the classical finite element method (FEM) usually produce sparse linear systems of equations $\varvec{A} \varvec{x} = \varvec{b}$ with storage requirements linear in the number of unknowns and linear complexity for the matrix-vector multiplication. However, the direct solution of these systems is computationally more expensive. Therefore, iterative solution methods (e.g., Krylov space methods) are popular in applications, since they only need matrix-vector multiplications, which can be done in linear complexity. A drawback of these methods is that convergence can be slow for matrices with large condition numbers unless a suitable preconditioner is employed. These preconditioners have to be taylored to the problem at hand making black box preconditioners that are based on (approximate) direct solvers particularly interesting. Moreover, if one is interested in solving the same problem with (many) different right-hand sides, a direct solver may be computationally advantageous.

Hierarchical matrices (${\mathcal{H}}$-matrices), introduced in [20] and extensively studied in the monograph [21], provide a different solution approach to this problem that does not suffer from the drawbacks of classical direct and iterative methods. ${\mathcal{H}}$-matrices are blockwise low-rank matrices. For suitable block structures and block ranks, storing an ${\mathcal{H}}$-matrix is of logarithmic-linear complexity. Approximating a given matrix in the ${\mathcal{H}}$-matrix format thus effects a compression. A main difference to other compression methods such as multipole expansions, [18, 23], or wavelet methods, [24–26], is that the ${\mathcal{H}}$-matrix format allows for an approximate arithmetic. It is possible to add and multiply as well as compute inverses and *LU*-decompositions efficiently in the format, [14, 19, 21]. Therefore, using an ${\mathcal{H}}$-matrix approximation to the inverse $\varvec{A}^{-1}$ gives an approximate direct solution method of logarithmic-linear complexity that can be applied efficiently to multiple right-hand sides. Moreover, an *LU*-decomposition in the ${\mathcal{H}}$-matrix format can be used as a black-box preconditioner in iterative solvers, [3, 15, 17, 22]. Nonetheless, we mention that the accuracy in terms of the maximal blockwise rank of the computed approximations to $\varvec{A}^{-1}$ (or the *LU*-decomposition) using ${\mathcal{H}}$-matrix arithmetic is not fully understood yet.

In order to explain the numerical success of these approximations, first observed in [19], several works in the literature provide existence results of approximations to the inverse matrices in the ${\mathcal{H}}$-matrix format. See, e.g., [2, 5, 7, 11] for the inverses of FEM matrices and [12, 13] for the inverses of BEM matrices. These analyses are restricted to the case of (quasi)uniform meshes, i.e., all mesh elements have comparable size. In a typical FEM scenario, however, locally refined meshes are employed with mesh elements varying greatly in size in order to account for effects such as locally reduced regularity of the solution. A classical example are graded meshes for the solution of elliptic problems in corner domains, [6].

In this article, we generalize the results of [11] for quasiuniform meshes to meshes of so called *locally bounded cardinality* (cf. Definition 2.4), which includes both uniform meshes and algebraically graded meshes. Our main result states that the inverses of FEM matrices for such meshes can be approximated by hierarchical matrices such that the error converges exponentially in the ${\mathcal{H}}$-matrix block rank *r*. Given a clustering strategy suitable for non-uniform grids, cf. [16], the storage complexity of the ${\mathcal{H}}$-matrix approximant is of logarithmic-linear complexity $\mathcal{O}(r N \ln N)$. Moreover, we develop an abstract framework that allows for more general FEM basis functions that do not need to have local supports. In fact, locality is necessary only for a set of *dual functions*, which is a substantially weaker assumption. Finally, we streamline some of the arguments made in [11]. While not repeated in this article, we mention that the (mostly algebraic) techniques of [11, Section 5] can be employed in exactly the same way to derive exponentially convergent approximate *LU*-decompositions in the ${\mathcal{H}}$-matrix format.

The present paper is structured as follows: In Sect. 2 we introduce all necessary definitions and concepts and state our main result, Theorem 2.15. Section 3 is dedicated to the proof of the main result. The main technical contribution is the discrete Caccioppoli-type estimate presented in Lemma 3.27, which is of independent interest. For a certain class of functions, it allows us to bound the $H^1$-seminorm on a given subdomain by the $L^2$-norm on a slightly larger subdomain. Finally, Sect. 4 provides numerical examples that illustrate our main result.

Concerning notation: We write “$a \lesssim b$”, if there exists a constant $C>0$ such that “$a \le C b$”. The constant might depend on the space dimension *d*, the domain $\varOmega$, the coefficients of the PDE, the shape regularity constant of the mesh, and the polynomial degree of the discrete spline space, but it is *in*dependent of all critical parameters such as the mesh width. We write $a \eqsim b$, if there hold both $a \lesssim b$ and $a \gtrsim b$. Matrices and vectors in linear systems of equations are expressed in boldface letters, e.g., $\varvec{A} \in \mathbb {R}^{N \times N}$ and $\varvec{f} \in \mathbb {R}^N$. For all $x \in \mathbb {R}^d$ and $\varepsilon > 0$, we write $\mathrm {Ball}_{2}(x,r) {:=} \{y \in \mathbb {R}^d\,|\,\Vert y-x\Vert _{2} < \varepsilon \}$ for the Euclidean ball of radius *r* centered at *x*. The norm of the sequence spaces $\ell ^1$ and $\ell ^2$ is denoted by $\Vert \cdot \Vert _{1}$ and $\Vert \cdot \Vert _{2}$. For $k \ge 0$, $q \in [1,\infty ]$ and domains $\varOmega \subseteq \mathbb {R}^d$, we denote the Sobolev space by $W^{k,q}(\varOmega )$. For a given mesh $\mathcal{T}$, we denote by $W^{k,q}_{\mathrm {pw}}(\mathcal{T}\,\,)$ the broken Sobolev space consisting of elementwise functions from $W^{k,q}$. For all $v \in W^{k,q}_{\mathrm {pw}}(\mathcal{T}\,\,)$ and $\mathcal{B}\subseteq \mathcal{T}$, we set $|v|_{W^{k,q}(\mathcal{B})} {:=} (\sum _{T \in \mathcal{B}} |v|_{W^{k,q}(T)}^q)^{1/q}$ and $|v|_{W^{k,\infty }(\mathcal{B})} {:=} \max _{T \in \mathcal{B}} |v|_{W^{k,\infty }(T)}$. Similarly, $C^{0}_{\mathrm {pw}}(\mathcal{T}\,\,)$ denotes the space of piecewise continuous functions. For all $v \in L^{2}(\varOmega )$ and $\mathcal{B}\subseteq \mathcal{T}$, the restriction of *v* to $\bigcup \mathcal{B}\subseteq \mathbb {R}^d$ is abbreviated as $v|_{\mathcal{B}} {:=} v|_{\bigcup \mathcal{B}}$. Finally, it will facilitate notation on numerous occasions to define the *(discrete) support* of a function $v \in L^{2}(\varOmega )$ on a mesh $\mathcal{T}$ by $\mathrm {supp}_{\mathcal{T}}\,(v) {:=} \{T \in \mathcal{T}\,|\,\,v|_{T} \not \equiv 0\}$. In particular, we have $\mathrm {supp}_{\mathcal{T}}\,(v) \subseteq \mathcal{T}$ and $\bigcup \mathrm {supp}_{\mathcal{T}}\,(v) \subseteq \mathbb {R}^d$, which slightly differs from the usual definition of a support, namely, $\mathrm {supp}_{}(v) {:=} \overline{\{x \in \varOmega \,|\,v(x) \ne 0\}} \subseteq \mathbb {R}^d$.

## Main results

### The model problem

We investigate the following *model problem*: Let $d \ge 1$ and $\varOmega \subseteq \mathbb {R}^d$ be a bounded polyhedral Lipschitz domain. Furthermore, let $a_1 \in L^{\infty }(\varOmega ,\mathbb {R}^{d \times d})$, $a_2 \in L^{\infty }(\varOmega ,\mathbb {R}^d)$ and $a_3 \in L^{\infty }(\varOmega ,\mathbb {R})$ be given coefficient functions and $f \in L^{2}(\varOmega )$ be a given right-hand side. We seek a weak solution $u \in H^{1}_{0}(\varOmega )$ to the following equations:

$$\begin{aligned}-\mathrm {div}_{}(a_1{\cdot}\nabla_{}u) + a_2 \cdot \nabla _{} u + a_3u &= f \quad \text {in} \,\, \varOmega , \\\quad u &= 0 \quad \text {on} \,\, \partial \varOmega . \end{aligned}$$

In the present work, we restrict ourselves to homogeneous Dirichlet conditions. For the treatment of Neumann and Robin boundary conditions, the same arguments as in [11] can be employed.

We assume that $a_1$ is coercive in the sense $\langle a_1(x) y,y\rangle _{} \ge \alpha _1 \Vert y\Vert _{2}^2$ for all $x \in \varOmega$, $y \in \mathbb {R}^d$ and some constant $\alpha _1 > \sigma _{\mathrm {Pcr}}^2 (\Vert a_2\Vert _{L^{\infty }(\varOmega )} + \Vert a_3\Vert _{L^{\infty }(\varOmega )}) \ge 0$. Here, $\sigma _{\mathrm {Pcr}}>0$ denotes the constant in the Poincaré inequality $\Vert \cdot \Vert _{H^{1}(\varOmega )} \le \sigma _{\mathrm {Pcr}}|\cdot |_{H^{1}(\varOmega )}$ on $H^{1}_{0}(\varOmega )$.

#### Definition 2.1

We introduce the bilinear form:

$$\begin{aligned} \forall u,v \in H^{1}_{0}(\varOmega ): \quad \quad a(u,v) {:=}\, \langle a_1 \nabla _{} u,\nabla _{} v\rangle _{L^{2}(\varOmega )} + \langle a_2 \,\cdot \,\nabla _{} u,v\rangle _{L^{2}(\varOmega )} + \langle a_3 u,v\rangle _{L^{2}(\varOmega )}. \end{aligned}$$

The weak formulation of the *model problem* reads as follows: Find $u \in H^{1}_{0}(\varOmega )$ such that

$$\begin{aligned} \forall v \in H^{1}_{0}(\varOmega ): \quad \quad a(u,v) = \langle f,v\rangle _{L^{2}(\varOmega )}. \end{aligned}$$

The assumptions on the PDE coefficients imply that the bilinear form $a(\cdot ,\cdot )$ is continuous and coercive, cf. Lemma 3.7. In particular, the well-known Lax-Milgram Lemma yields the existence of a unique solution $u \in H^{1}_{0}(\varOmega )$.

### The mesh

Throughout the text, we consider regular, affine meshes in the following sense:

#### Definition 2.2

*(Mesh)* A finite set $\mathcal{T}\subseteq \mathrm {Pow}(\varOmega )$ is a *mesh* if there exists an open simplex $\hat{T}\subseteq \mathbb {R}^d$ (the *reference element*) such that every *element* $T \in \mathcal{T}$ is of the form $T = F_T(\hat{T})$, where $F_T: \mathbb {R}^d \longrightarrow \mathbb {R}^d$ is an affine diffeomorphism. Furthermore, the elements must be pairwise disjoint, i.e., $|T \cap S|_{} = 0$ for all $T \ne S \in \mathcal{T}$, and constitute a partition of $\varOmega$, i.e., $\bigcup _{T \in \mathcal{T}} \overline{T} = \overline{\varOmega }$. Finally, a mesh must be regular in the sense of [9], i.e., it does not contain any hanging nodes.

We call a collection of mesh elements $\mathcal{B}\subseteq \mathcal{T}$ a *cluster*. In the literature on hierarchical matrices, the word *cluster* is typically reserved for collections of vector/matrix indices $I \subseteq \{1,\dots ,N\}$. In the present work, however, we deal with collections of mesh elements $\mathcal{B}\subseteq \mathcal{T}$ much more frequently. We also note that both concepts are intimately linked via Definition 2.8.

For every subset $B \subseteq \mathbb {R}^d$, we call the set of neighboring mesh elements

2.1 $$\begin{aligned} \mathcal{T}(B) {:=} \{T \in \mathcal{T}\,|\,\overline{T} \cap \overline{B} \ne \emptyset \} \subseteq \mathcal{T}\end{aligned}$$

the *patch* of *B*. Similarly, for every cluster $\mathcal{B}\subseteq \mathcal{T}$, we set $\mathcal{T}(\mathcal{B}) {:=} \bigcup _{B \in \mathcal{B}} \mathcal{T}(B) \subseteq \mathcal{T}$.

To measure the size of an element $T \in \mathcal{T}$, we introduce the local *mesh width* $h_{T} {:=} \sup _{x,y \in T} \Vert y-x\Vert _{2}$. The corresponding aggregate mesh widths for a cluster $\mathcal{B}\subseteq \mathcal{T}$ read $h_{\mathcal{B}} {:=} h_{\max ,\mathcal{B}} {:=} \max _{T \in \mathcal{B}} h_{T}$ and $h_{\min ,\mathcal{B}} {:=} \min _{T \in \mathcal{B}} h_{T}$.

Finally, for every $T \in \mathcal{T}$, we denote the center of the largest inscribable ball by $x_T \in T$ (the *incenter*). We assume that $\mathcal{T}$ is part of a *shape-regular* family of meshes, i.e., there exists a constant $\sigma _{\mathrm {shp}}\ge 1$ such that

$$\begin{aligned} \forall T \in \mathcal{T}: \quad \quad \mathrm {Ball}_{2}(x_T,\sigma _{\mathrm {shp}}^{-1} h_{T}) \subseteq T \subseteq \bigcup \mathcal{T}(T) \subseteq \mathrm {Ball}_{2}(x_T,\sigma _{\mathrm {shp}}h_{T}). \end{aligned}$$

#### Definition 2.3

*(Mesh metric)* The *mesh metric* is given by

$$\begin{aligned} \forall T,S \in \mathcal{T}: \quad \quad \mathrm {dist}_{\mathcal{T}}\,(T,S) {:=} \Vert x_S-x_T\Vert _{2}. \end{aligned}$$

For all clusters $\mathcal{A}$, $\mathcal{B}\subseteq \mathcal{T}$, we denote the corresponding diameters and distances by

$$\begin{aligned} \mathrm {diam}_{\mathcal {T}}\,(\mathcal {A}) {:=}\,\max _{A_1,\;A_2 \in \mathcal {A}} \mathrm {dist}_{\mathcal {T}}\;(A_1,A_2), \quad \quad \mathrm {dist}_{\mathcal {T}}\;(\mathcal {A},\mathcal {B}) {:=} \min _{\begin{subarray}{c} A \in \mathcal {A}, \\ B \in \mathcal {B} \end{subarray}} \mathrm {dist}_{\mathcal {T}}\;(A,B). \end{aligned}$$

If $\mathcal{A}$ or $\mathcal{B}$ contains only one element, e.g., $\mathcal{A}= \{T\}$, we drop the enclosing braces and simply write $\mathrm {dist}_{\mathcal{T}}\,(T,\mathcal{B}) {:=} \mathrm {dist}_{\mathcal{T}}\,(\{T\},\mathcal{B})$. Furthermore, $\mathrm {diam}_{\mathcal{T}}\,(T) {:=} \mathrm {diam}_{\mathcal{T}}\,(\{T\}) = 0$ by definition of the cluster diameter.

We refer to Lemma 3.15 for some basic properties of the mesh metric.

Compared to [11], we consider a more general class of meshes. Here, the crucial property is the so called *locally bounded cardinality* defined in the following Definition 2.4. In Sect. 3.2, we provide examples of meshes both with (uniform and algebraically graded meshes) and without said property (exponentially graded meshes).

#### Definition 2.4

*(Locally bounded cardinality)* A mesh $\mathcal{T}\subseteq \mathrm {Pow}(\varOmega )$ has *locally bounded cardinality*, if there exists a constant $\sigma _{\mathrm {card}}\ge 1$ such that

$$\begin{aligned} h_{\mathcal{T}}^{\sigma _{\mathrm {card}}} \lesssim h_{\min ,\mathcal{T}}, \quad \quad \quad \forall \mathcal{B}\subseteq \mathcal{T}: \quad \# \mathcal{B} \lesssim \bigg (1 + \frac{\mathrm {diam}_{\mathcal{T}}(\mathcal{B})}{h_{\mathcal{B}}} \bigg )^{d\sigma _{\mathrm {card}}}. \end{aligned}$$

### The basis and dual functions

#### Definition 2.5

*(Spline spaces)* Let $k \ge 0$ and $p \ge 0$. We introduce the finite-dimensional *spline spaces*

$$\begin{aligned} \mathbb {S}^{p,k}(\mathcal{T}\,\,){:=}&\{v \in H^{k}(\varOmega )\,|\,\forall T \in \mathcal{T}: v \circ F_T \in \mathbb {P}^{p}(\hat{T})\}, \\ \mathbb {S}^{p,k}_{0}(\mathcal{T}\;){:=}&\mathbb {S}^{p,k}(\mathcal{T}\;) \cap H^{1}_{0}(\varOmega ), \end{aligned}$$

where $\mathbb {P}^{p}(\hat{T}) {:=} \mathrm {span}\,\{\hat{T}\ni x \mapsto x^q\,|\,\Vert q\Vert _{1} \le p\}$ denotes the usual space of polynomials of (total) degree *p* on the reference element.

The following definition introduces the bases of $\mathbb {S}^{p,1}_{0}(\mathcal{T}\,\,)$ that we consider:

#### Definition 2.6

*(Basis with local dual functions)* Let $p \ge 1$, $N {:=} \mathrm {dim}\,\mathbb {S}^{p,1}_{0}(\mathcal{T}\,\,)$ and $\{\varphi _1,\dots ,\varphi _N\} \subseteq \mathbb {S}^{p,1}_{0}(\mathcal{T}\,\,)$ be a basis. We say that the basis *allows for a system of local dual functions*, if there exist functions $\{\lambda _1,\ldots ,\lambda _N\} \subseteq L^{2}(\varOmega )$ with the following properties:

1. Duality: For all $n,m \in \{1,\dots ,N\}$, there holds $\langle \varphi _n,\lambda _m\rangle _{L^{2}(\varOmega )} = \delta _{nm}$.
2. Stability: For all $\varvec{x} \in \mathbb {R}^N$, there holds the bound $\Vert \sum _{m=1}^{N} \varvec{x}_m \lambda _m\Vert _{L^{2}(\varOmega )} \lesssim h_{\min ,\mathcal{T}}^{-d/2} \Vert \varvec{x}\Vert _{2}$. The implied constant may only depend on *d*, *p*, and the shape regularity of the mesh $\mathcal{T}$.
3. Locality: For every $n \in \{1,\dots ,N\}$, there exists a characteristic element $T_n \in \mathcal{T}$ such that $\mathrm {supp}_{\mathcal{T}}(\lambda _n)\subseteq \mathcal{T}(T_n)$. For every $T \in \mathcal{T}$, there holds the uniform bound $\# \{n\,|\,T_n = T\} \lesssim 1$.

#### Remark 2.7

Note that we *do not* assume *local* basis functions $\varphi _n$, i.e., $\mathrm {supp}_{\mathcal{T}}\,(\varphi _n) = \mathcal{T}$ is allowed. Rather, locality is imposed only on the dual functions. In the *finite element* framework described in Sect. 3.3, this distinction might seem unnecessary, as both the basis functions and the dual functions are indeed local. In the somewhat similar setting of *radial basis functions* (see, e.g., [27]), however, the distinction becomes crucial. There, the basis functions have global supports by nature and locality can only be imposed on the dual functions. Here, our goal is to formulate the more general framework such that we can apply some results in our upcoming work on ${\mathcal{H}}$-matrices and radial basis functions (cf. [1]) as well.

The fundamental idea of the present work is to derive properties of matrices from properties of function spaces. Naturally, one has to think about the connection between abstract matrix indices $n \in \{1,\ldots ,N\}$ and corresponding subdomains of $\varOmega$, which is captured in the following definition.

#### Definition 2.8

*(Index patches)* We define the *index patches*

$$\begin{aligned} \forall I \subseteq \{1,\dots ,N\}: \quad \quad \mathcal{T}(I) {:=} \bigcup _{n \in I} \mathrm {supp}_{\mathcal{T}}\,(\lambda _n) \subseteq \mathcal{T}. \end{aligned}$$

Recall from Sect. 2.2 that $\mathcal{T}(B) \subseteq \mathcal{T}$ is the patch of a subdomain $B \subseteq \mathbb {R}^d$ and that $\mathcal{T}(\mathcal{B}) \subseteq \mathcal{T}$ is the patch of a cluster $\mathcal{B}\subseteq \mathcal{T}$. We also have patches $\mathcal{T}(I) \subseteq \mathcal{T}$ for collections of matrix indices $I \subseteq \{1,\dots ,N\}$. Since all three types of patches follow a common idea, we chose the similarity in notation on purpose.

### The system matrix

Let $\mathcal{T}\subseteq \mathrm {Pow}(\varOmega )$ be a mesh and $p \ge 1$ a fixed polynomial degree. Let $\mathbb {S}^{p,1}_{0}(\mathcal{T}\,\,) \subseteq H^{1}_{0}(\varOmega )$ be the corresponding spline space. We discretize the model problem from Sect. 2.1 by means of the spline space and get the following *discrete model problem*: For given $f \in L^{2}(\varOmega )$, find $u \in \mathbb {S}^{p,1}_{0}(\mathcal{T}\,\,)$ such that

$$\begin{aligned} \forall v \in \mathbb {S}^{p,1}_{0}(\mathcal{T}\,\,): \quad \quad a(u,v) = \langle f,v\rangle _{L^{2}(\varOmega )}. \end{aligned}$$

Again, existence and uniqueness of a solution $u \in \mathbb {S}^{p,1}_{0}(\mathcal{T}\,\,)$ follow from Lemma 3.7 and the Lax-Milgram Lemma.

As usual, given a basis of the discrete space, the discrete model problem can be rephrased as an equivalent linear system of equations. The bilinear form $a(\cdot ,\cdot )$ from Definition 2.1 and the basis functions $\varphi _n \in \mathbb {S}^{p,1}_{0}(\mathcal{T}\,\,)$ from Definition 2.6 compose the governing system matrix.

#### Definition 2.9

We define the system matrix

$$\begin{aligned} \varvec{A} {:=} \,(a(\varphi _n,\varphi _m))_{m,n=1}^{N} \in \mathbb {R}^{N \times N}. \end{aligned}$$

Note that the unique solvability of the discrete model problem already ensures that the matrix $\varvec{A}$ is invertible.

### Hierarchical matrices

First, let us sketch the concepts of *cluster trees* and *block cluster trees*. For a more detailed introduction, see, e.g., [21, Chapter 5].

A *(binary) clustering strategy* consists of two mappings $\mathrm {child}_1$, $\mathrm {child}_2: \mathrm {Pow}\{1,\dots ,N\} \longrightarrow \mathrm {Pow}\{1,\dots ,N\}$ that satisfy the disjointness property $\mathrm {child}_1(I) \cap \mathrm {child}_2(I) = \emptyset$ and the covering property $\mathrm {child}_1(I) \cup \mathrm {child}_2(I) = I$. (See, e.g., [21] for some examples of such clustering strategies.) Let $I_{\mathrm {root}} {:=} \{1,\dots ,N\}$ and $\sigma _{\mathrm {small}}> 0$. Consider a system $\mathbb {T}_{N}^{} \subseteq \mathrm {Pow}\{1,\dots ,N\}$ with $I_{\mathrm {root}} \in \mathbb {T}_{N}^{}$ that is closed in the following sense: For every tree node $I \in \mathbb {T}_{N}^{}$ with $\# I > \sigma _{\mathrm {small}}$, its children satisfy $\mathrm {child}_1(I)$, $\mathrm {child}_2(I) \in \mathbb {T}_{N}^{}$ as well. If $\mathbb {T}_{N}^{}$ is minimal, i.e., removing one of its elements would violate said properties, then we call $\mathbb {T}_{N}^{}$ a *(hierarchical) cluster tree*. The assumed minimality allows us to assign a *level* to each tree node such that $\mathrm {level}(I_{\mathrm {root}}) = 0$ and $\mathrm {level}(\mathrm {child}_1(I)) = \mathrm {level}(\mathrm {child}_2(I)) = \mathrm {level}(I) + 1$ for all $I \in \mathbb {T}_{N}^{}$. Finally, we set $\mathrm {depth}(\mathbb {T}_{N}^{}) {:=} \max _{I \in \mathbb {T}_{N}^{}} \mathrm {level}(I)$.

Said clustering strategy induces functions

$$\mathrm {child}_{11},\, \mathrm {child}_{12}, \, \mathrm {child}_{21}, \, \mathrm {child}_{22}: (\mathrm {Pow}\{1,\dots ,N\})^2 \longrightarrow (\mathrm {Pow}\{1,\dots ,N\})^2$$

via $\mathrm {child}_{\alpha \beta }(I,J) {:=} (\mathrm {child}_{\alpha }(I), \mathrm {child}_{\beta }(J))$. Let $\sigma _{\mathrm {adm}}> 0$. Consider a system $\mathbb {T}_{N \times N}^{} \subseteq (\mathrm {Pow}\{1,\dots ,N\})^2$ with $(I_{\mathrm {root}}, I_{\mathrm {root}}) \in \mathbb {T}_{N \times N}^{}$ that is closed in the following sense: For every tree node $(I,J) \in \mathbb {T}_{N \times N}^{}$ with $\mathrm {diam}_{\mathcal{T}}\,(\mathcal{T}(I)) > \sigma _{\mathrm {adm}}\mathrm {dist}_{\mathcal{T}}\,(\mathcal{T}(I),\mathcal{T}(J))$, its children satisfy $\mathrm {child}_{11}(I,J)$, $\mathrm {child}_{12}(I,J)$, $\mathrm {child}_{21}(I,J)$, $\mathrm {child}_{22}(I,J) \in \mathbb {T}_{N \times N}^{}$ as well. Again, if $\mathbb {T}_{N \times N}^{}$ is minimal, it is called *(hierarchical) block cluster tree*. The associated *sparsity constant* is given by

$$\begin{aligned} C_{\mathrm {sparse}}(\mathbb {T}_{N \times N}^{}) {:=}  \max \bigg \{\max _{I \in \mathbb {T}_{N}^{}} \# \{J \in \mathbb {T}_{N}^{}\,|\,(I,J) \in \mathbb {T}_{N \times N}^{}\}, \,\, \max _{J \in \mathbb {T}_{N}^{}} \# \{I \in \mathbb {T}_{N}^{}\,|\,(I,J) \in \mathbb {T}_{N \times N}^{}\}\bigg \}. \end{aligned}$$

#### Definition 2.10

The subset $\mathbb {P}\subseteq \mathbb {T}_{N \times N}^{}$ of all block cluster tree leaves is called *hierarchical block partition*. We say that $\mathbb {P}$ is *sparse*, if $\mathrm {depth}(\mathbb {T}_{N}^{}) \lesssim \ln (N)$ and $C_{\mathrm {sparse}}(\mathbb {T}_{N \times N}^{}) \lesssim 1$.

#### Remark 2.11

For a mesh $\mathcal{T}$ with locally bounded cardinality and a basis $\{\varphi _1,\dots ,\varphi _N\} \subseteq \mathbb {S}^{p,1}_{0}(\mathcal{T}\,\,)$ with local dual functions $\{\lambda _1,\dots ,\lambda _N\} \subseteq L^{2}(\varOmega )$, there indeed exists a *sparse* hierarchical block partition: The locality assumption on the dual functions allows us to treat their supports as a group of distinct characteristic points in $\mathbb {R}^d$. Thus, we can apply the results from [16]. There, the authors presented a *geometrically balanced* clustering strategy that ensures the upper bounds $\mathrm {depth}(\mathbb {T}_{N}^{}) \lesssim \ln (h_{\min ,\mathcal{T}}^{-1})$ and $C_{\mathrm {sparse}}(\mathbb {T}_{N \times N}^{}) \lesssim 1$ for the resulting trees $\mathbb {T}_{N}^{}$ and $\mathbb {T}_{N \times N}^{}$. Using the relation $h_{\min ,\mathcal{T}} \gtrsim h_{\mathcal{T}}^{\sigma _{\mathrm {card}}}$ from Definition 2.4 for meshes with locally bounded cardinality, we conclude $\mathrm {depth}(\mathbb {T}_{N}^{}) \lesssim \ln (N)$, i.e., the hierarchical block partition $\mathbb {P}$ is sparse.

From the construction of the block cluster tree it follows that the elements of the hierarchical block partition can be categorized into two groups. More precisely, we can state the following:

#### Lemma 2.12

*The hierarchical block partition can be decomposed as* $\mathbb {P}= \mathbb {P}_{\mathrm {adm}}\,\, \dot{\cup } \,\, \mathbb {P}_{\mathrm {small}}$ *with*

$$\begin{aligned} \begin{array}{lrclcl} \forall (I,J) \in \mathbb {P}_{\mathrm {adm}}: &{} 0 &{}<&{} \mathrm {diam}_{\mathcal{T}}\,(\mathcal{T}(I)) &{}\le &{} \sigma _{\mathrm {adm}}\mathrm {dist}_{\mathcal{T}}\,(\mathcal{T}(I),\mathcal{T}(J)), \\ \forall (I,J) \in \mathbb {P}_{\mathrm {small}}: \quad &{} &{}&{} \min \{\# I, \# J\} &{}\le &{} \sigma _{\mathrm {small}}. \end{array} \end{aligned}$$

#### Definition 2.13

Let $\mathbb {P}$ be a sparse hierarchical block partition and $r \in \mathbb {N}$ a given *block rank bound*. We define the set of ${\mathcal{H}}$-*matrices* by

$$\begin{aligned} {\mathcal{H}}(\mathbb {P},r) {:=} \{\varvec{B} \in \mathbb {R}^{N \times N}\,|\,\forall (I,J) \in \mathbb {P}_{\mathrm {adm}}: \exists \varvec{X} \in \mathbb {R}^{I \times r}, \varvec{Y} \in \mathbb {R}^{J \times r}: \varvec{B}|_{I \times J} = \varvec{X} \varvec{Y}^T\}. \end{aligned}$$

#### Remark 2.14

By [21, Lemma 6.13], the memory requirement to store an ${\mathcal{H}}$-matrix $\varvec{B} \in {\mathcal{H}}(\mathbb {P},r)$ can be bounded by the quantity $C_{\mathrm {sparse}}(\mathbb {T}_{N \times N}^{}) (\sigma _{\mathrm {small}}+ r) \mathrm {depth}(\mathbb {T}_{N}^{}) N$. Inserting the estimates for the cluster tree depth and the sparsity constant of a *sparse* hierarchical block partition (cf. Definition 2.10), we get an overall bound of $\mathcal{O}(r N \ln N)$ for the memory requirement.

### The main result

The following theorem is the main result of the present work. It states that inverses of FEM matrices with meshes of locally bounded cardinality can be approximated at an exponential rate in the block rank by hierarchical matrices.

#### Theorem 2.15

*Let* $\mathcal{T}\subseteq \mathrm {Pow}(\varOmega )$ *be a mesh of locally bounded cardinality for some* $\sigma _{\mathrm {card}}\ge 1$ *in the sense of Definition* 2.4*and* $\{\varphi _1,\dots ,\varphi _N\} \subseteq \mathbb {S}^{p,1}_{0}(\mathcal{T}\,\,)$ *a basis that allows for a system of local dual functions* (*see Definition* 2.6). *Let* $a(\cdot ,\cdot )$ *be the elliptic bilinear form from* *Definition* 2.1*and* $\varvec{A} \in \mathbb {R}^{N \times N}$ *be the corresponding Galerkin stiffness matrix* (*Definition* 2.9). *Finally, let* $\mathbb {P}$ *be a sparse hierarchical block partition as in Definition* 2.10. *Then, there exists a constant* $\sigma _{\mathrm {exp}}>0$ *such that, for every block rank bound* $r \in \mathbb {N}$, *there exists an* ${\mathcal{H}}$-*matrix* $\varvec{B} \in {\mathcal{H}}(\mathbb {P},r)$ *with*

$$\begin{aligned} \Vert \varvec{A}^{-1} - \varvec{B}\Vert _{2} \lesssim N^{\sigma _{\mathrm {card}}}\ln (N) \exp (-\sigma _{\mathrm {exp}}r^{1/(d\sigma _{\mathrm {card}}+ 1)}). \end{aligned}$$

#### Remark 2.16

Comparing the main result with the previous work [11] shows that the parameter $\sigma _{\mathrm {card}}$ of a mesh with locally bounded cardinality additionally appears. For quasi-uniform meshes, as studied in [11], this parameter is given by $\sigma _{\mathrm {card}}= 1$ and Theorem 2.15 reproduces the main result therein. However, for different meshes such as algebraically graded meshes, this parameter reduces the exponential rate of convergence. With our fully discrete method of proof a dependence on mesh parameters can be expected and seems unavoidable. Nonetheless, a numerical example presented at the end of the paper shows that the rate of convergence indeed depends on $\sigma _{\mathrm {card}}$, albeit the dependence seems to be weaker than in the theoretical bound of Theorem 2.15.

As shown in Sect. 3.2, *uniform* and *algebraically graded* meshes have locally bounded cardinality. In particular, we immediately get the following corollary.

#### Corollary 2.17

*Let* $\mathcal{T}\subseteq \mathrm {Pow}(\varOmega )$ *be an algebraically graded mesh with grading exponent* $\alpha \ge 1$ (*see Definition* 3.4). *Then*, *Theorem* 2.15*holds verbatim with* $\sigma _{\mathrm {card}}= \alpha$.

## Proof of main result

### Overview

The techniques employed in the proof of our main result are similar to those developed in [11] for uniform meshes. However, some modifications are necessary to deal with the present case of non-uniform meshes $\mathcal{T}$ and (possibly) global basis functions $\varphi _n \in \mathbb {S}^{p,1}_{0}(\mathcal{T}\,\,)$. Additionally, we simplify several parts of the previous proof considerably.

(1) Before we begin the proof, we give a motivation for the assumptions made in Definitions 2.4 and 2.6. In Sect. 3.2, we present two types of meshes with locally bounded cardinality, namely *uniform* and *algebraically graded* meshes. The fact that every uniform mesh has locally bounded cardinality will be used during our proof of Theorem 3.31. The locally bounded cardinality of algebraically graded meshes shows that Theorem 2.15 is applicable for algebraically graded meshes in the sense of Definition 3.4.

Then, in Sect. 3.3, we present a practical choice for the dual functions $\lambda _n \in L^{2}(\varOmega )$ from Definition 2.6 for a common choice of basis functions $\varphi _n \in \mathbb {S}^{p,1}_{0}(\mathcal{T}\,\,)$. The results from this section guarantee that Theorem 2.15 can be used for many different types of *finite element* bases, including the classic *hat functions*.

(2) The starting point for our proof is an explicit representation formula for $\varvec{A}^{-1}$. Since $\varvec{A}^{-1}$ represents the act of solving the discretized model problem, it is only natural that the corresponding *discrete solution operator* $S_{\mathcal{T}}: L^{2}(\varOmega ) \longrightarrow \mathbb {S}^{p,1}_{0}(\mathcal{T}\,\,)$ will be involved. Additionally, this endeavor requires the dual functions $\lambda _n \in L^{2}(\varOmega )$ mentioned earlier. We present the explicit formula for $\varvec{A}^{-1}$ at the end of Sect. 3.4.

(3) In Sect. 3.5 we use this formula to go from the “matrix level” to the “function level”: Initially, we reduce the problem of approximating $\varvec{A}^{-1}$ as a whole to the problem of approximating $\varvec{A}^{-1}|_{I \times J}$ for each admissible block $(I,J) \in \mathbb {P}_{\mathrm {adm}}$. (The small blocks $\mathbb {P}_{\mathrm {small}}$ are irrelevant for that matter.) As it turns out, this boils down to the following question:

Given admissible clusters $\mathcal{B}, \mathcal{D}\subseteq \mathcal{T}$ and a free parameter $L \in \mathbb {N}$, how can we construct a low-dimensional subspace $V_{\mathcal{B},\mathcal{D},L} \subseteq L^{2}(\varOmega )$ that contains a good approximant of $(S_{\mathcal{T}} \,f)|_{\mathcal{B}}$ for every $f \in L^{2}(\varOmega )$ with $\mathrm {supp}_{\mathcal{T}}\,(f) \subseteq \mathcal{D}$? More precisely, we want to achieve the bounds (for some fixed $\kappa \ge 1$)

$$\begin{aligned} \mathrm {dim}\,V_{\mathcal{B},\mathcal{D},L} \lesssim L^{\kappa }, \quad \quad \quad \inf _{v \in V_{\mathcal{B},\mathcal{D},L}} \Vert S_{\mathcal{T}}\, f - v\Vert _{L^{2}(\mathcal{B})} \lesssim 2^{-L} \Vert f\Vert _{L^{2}(\mathcal{D})}. \end{aligned}$$

The remaining sections will give an answer to this very question. Since the construction of $V_{\mathcal{B},\mathcal{D},L}$ is fairly technical and by no means straightforward, the proof is split into further parts:

(4) As the notation “$V_{\mathcal{B},\mathcal{D},L}$” already suggests, the notion of *locality* plays a prominent role in almost all parts of the proof. This is why we introduce so called *inflated clusters*, *discrete cut-off functions*, and the *discrete cut-off operator* in Sect. 3.6.

(5) In Sect. 3.7 we investigate an important class of functions for our analysis, the spaces of *locally discrete harmonic functions* $\mathbb {S}_{\mathrm {harm}}(\mathcal{B}) \subseteq \mathbb {S}^{p,1}_{0}(\mathcal{T}\,\,)$. These subspaces have three important properties: First, for certain $f \in L^{2}(\varOmega )$, they contain the image $S_{\mathcal{T}} \,f$. Second, they are invariant under the influence of their respective discrete cut-off operators. Third, they allow for the *discrete Caccioppoli inequality*, a key ingredient in deriving the asserted error bounds for $V_{\mathcal{B},\mathcal{D},L}$.

(6) Finally, in Sect. 3.8 we construct the *single-* and *multi-step coarsening operators*. For any given $u \in \mathbb {S}_{\mathrm {harm}}({\mathcal{B}}^{\delta }_{})$ on the inflated cluster ${\mathcal{B}}^{\delta }_{} \supseteq \mathcal{B}$, the single-step coarsening operator $Q_{\mathcal{B}}^{\delta }$ produces a “coarse” approximation $Q_{\mathcal{B}}^{\delta } u \in \mathbb {S}_{\mathrm {harm}}(\mathcal{B})$ with a small approximation error on $\mathcal{B}$. This is by far the most intricate part of the proof and puts all the aforementioned concepts to use. Afterwards, the multi-step coarsening operator $Q_{\mathcal{B}}^{\delta ,L}$ is just a combination of $L \in \mathbb {N}$ single-step coarsening operators.

(7) In Sect. 3.9 we merely put all the pieces together and finish the proof of Theorem 2.15.

### Examples of meshes with locally bounded cardinality

In this subsection, we present two representatives of meshes with locally bounded cardinality (cf. Definition 2.4): *Uniform* meshes and *algebraically graded* meshes. To verify the locally bounded cardinality property for a given mesh, the following lemma is helpful.

#### Lemma 3.1

*Let* $\mathcal{T}\subseteq \mathrm {Pow}(\varOmega )$ *be a shape-regular mesh as in Definition* 2.2. *Then, there hold the bounds*

$$\begin{aligned} \frac{1}{h_{\mathcal{T}}^d} \lesssim \# \mathcal{T}, \quad \quad \quad \forall \mathcal{B}\subseteq \mathcal{T}: \quad \# \mathcal{B} \lesssim \bigg (1 + \frac{\mathrm {diam}_{\mathcal{T}}\,(\mathcal{B})}{h_{\min ,\mathcal{B}}} \bigg )^d. \end{aligned}$$

#### Proof

The first relation follows immediately from $1 \eqsim |\varOmega |_{} = \sum _{T \in \mathcal{T}} |T|_{} \eqsim \sum _{T \in \mathcal{T}} h_{T}^d \le h_{\mathcal{T}}^d \#\mathcal{T}$. For the second bound, let $\mathcal{B}\subseteq \mathcal{T}$. Using Lemma 3.15 ahead, we get $h_{\min ,\mathcal{B}}^d \# \mathcal{B} \le \sum _{T \in \mathcal{B}} h_{T}^d \eqsim \sum _{T \in \mathcal{B}} |T|_{} = |\bigcup \mathcal{B}|_{} \lesssim (\mathrm {diam}_{\mathcal{T}}\,(\mathcal{B}) + h_{\mathcal{B}})^d \lesssim (\mathrm {diam}_{\mathcal{T}}\,(\mathcal{B}) + h_{\min ,\mathcal{B}})^d$. This concludes the proof. $\square$

#### Definition 3.2

*(Uniform mesh)* A mesh $\mathcal{T}\subseteq \mathrm {Pow}(\varOmega )$ is called *uniform*, if there exists a constant $\sigma _{\mathrm {unif}}\ge 1$ such that

$$\begin{aligned} h_{\min ,\mathcal{T}} \le h_{\mathcal{T}} \le \sigma _{\mathrm {unif}}h_{\min ,\mathcal{T}}. \end{aligned}$$

Using Lemma 3.1, we immediately get the following result:

#### Lemma 3.3

*Every uniform mesh* $\mathcal{T}\subseteq \mathrm {Pow}(\varOmega )$ *has locally* *bounded cardinality with* $\sigma _{\mathrm {card}}= 1$.

#### Definition 3.4

(*Mesh graded towards* $\Gamma$) Let $\mathcal{T}\subseteq \mathrm {Pow}(\varOmega )$ be a mesh and $\Gamma \subseteq \mathbb {R}^d$ satisfy $\Gamma \subseteq \mathbb {R}^d \backslash T$ for all $T \in \mathcal{T}$. Furthermore, let $\alpha \ge 1$ be a *grading exponent* and $H>0$ a *coarse mesh width*. We say that $\mathcal{T}$ is *(algebraically) graded towards* $\Gamma$ *with parameters* $\alpha ,H$, if there holds

$$\begin{aligned} \forall T \in \mathcal{T}: \quad \quad h_{T} \eqsim \mathrm {dist}_{2}(x_T,\Gamma )^{1-1/\alpha } H. \end{aligned}$$

Here, $x_T$ denotes the incenter of the element *T* and $\mathrm {dist}_{2}(x_T,\Gamma ) =  \inf _{\gamma \in \Gamma } \Vert x_T-\gamma \Vert _{2}$ is the Euclidean distance between a point and a set.

The set $\Gamma$, towards which the mesh is graded, is usually determined by the given problem. For example, reentrant corners of the domain $\varOmega$ or regions of non-smoothness of the data may entail a reduced regularity of the solution *u* to the model problem from Sect. 2.1. This usually leads to reduced order of convergence of the finite element approximation on quasiuniform meshes. Choosing the set $\Gamma$ to contain all singularities of the solution as well as choosing the parameter $\alpha$ correctly, one can restore the optimal order of convergence. To a large extent, the shape of $\Gamma$ is irrelevant for our analysis. We only require that the mesh resolves $\Gamma$, i.e., the mesh can only be graded towards subsets of the mesh skeleton, e.g., vertices/edges/faces in 3D.

#### Lemma 3.5

*Let* $\mathcal{T}\subseteq \mathrm {Pow}(\varOmega )$ *be a mesh graded towards* $\Gamma$ *with parameters* $\alpha ,H$. *Then, there hold the bounds* $H^{\alpha } \lesssim h_{\min ,\mathcal{T}} \le h_{\mathcal{T}} \lesssim H$. *Furthermore*, $\mathcal{T}$ *has locally bounded cardinality with* $\sigma _{\mathrm {card}}= \alpha$.

#### Proof

We start with the bounds for $h_{\mathcal{T}}$ and $h_{\min ,\mathcal{T}}$: For every $T \in \mathcal{T}$, we know from Definition 2.2 that $\mathrm {Ball}_{2}(x_T,\sigma _{\mathrm {shp}}^{-1} h_{T}\,\,) \subseteq T$. Combining this with the assumption $\Gamma \subseteq T^c$ from Definition 3.4 yields $\mathrm {dist}_{2}(x_T,\Gamma ) \ge h_{T}/\sigma _{\mathrm {shp}}$. We conclude $h_{T} \eqsim \mathrm {dist}_{2}(x_T,\Gamma )^{1-1/\alpha } H \gtrsim h_{T}^{1-1/\alpha } H$ and ultimately $h_{\min ,\mathcal{T}} \gtrsim H^{\alpha }$. On the other hand, we have the bound $h_{T} \eqsim \mathrm {dist}_{2}(x_T,\Gamma )^{1-1/\alpha } H \le \sup _{x \in \varOmega } \mathrm {dist}_{2}(x,\Gamma )^{1-1/\alpha } H \lesssim H$ and thus $h_{\mathcal{T}} \lesssim H$.

It remains to prove the locally bounded cardinality: Let $\mathcal{B}\subseteq \mathcal{T}$ be arbitrary. We fix an element $B \in \mathcal{B}$ with $b {:=} \mathrm {dist}_{2}(x_B,\Gamma ) = \min _{T \in \mathcal{B}} \mathrm {dist}_{2}(x_T,\Gamma )$ and abbreviate $\Delta b {:=}\, \mathrm {diam}_{\mathcal{T}}\,(\mathcal{B})$. Note that $h_{\mathcal{B}} \eqsim (\max _{T \in \mathcal{B}} \mathrm {dist}_{2}(x_T,\Gamma ))^{1-1/\alpha } H \lesssim (b + \Delta b)^{1-1/\alpha } H$.

In the case $b \le \Delta b$ we have the lower bound

$$\begin{aligned} h_{\min ,\mathcal{B}} \ge h_{\min ,\mathcal{T}} \gtrsim H^{\alpha } \gtrsim \frac{h_{\mathcal{B}}^{\alpha }}{(b+\Delta b)^{\alpha -1}} \ge \frac{h_{\mathcal{B}}^{\alpha }}{(2\Delta b)^{\alpha -1}}. \end{aligned}$$

In the remaining case $b > \Delta b$ we get

$$\begin{aligned} h_{\min ,\mathcal{B}} \eqsim H \bigg (\min _{T \in \mathcal{B}} \mathrm {dist}_{2}(x_T,\Gamma ) \bigg )^{1-1/\alpha } = H b^{1-1/\alpha } \gtrsim h_{\mathcal{B}} \bigg (\frac{b}{b+\Delta b} \bigg )^{1-1/\alpha } \ge 2^{1/\alpha -1} h_{\mathcal{B}}. \end{aligned}$$

In particular, both cases lead to the estimate

$$\begin{aligned} \# \mathcal{B} {\mathop {\lesssim }\limits ^{\text {Lem.~3.1}}} \bigg (1 + \frac{\Delta b}{h_{\min ,\mathcal{B}}} \bigg )^d \lesssim \bigg (1 + \frac{\Delta b}{h_{\mathcal{B}}} \bigg )^{d\alpha }, \end{aligned}$$

which concludes the proof. $\square$

In order to gain a better understanding of Definition 2.4 it is instructive to investigate a counter example as well. To this end, let $\varOmega {:=} (0,1) \subseteq \mathbb {R}$, $M \in \mathbb {N}$, $\xi _m {:=} 2^{-m}$ for all $m \in \{0,\dots ,M-1\}$ and $\xi _M {:=} 0$. Then, the elements $T_m {:=} (\xi _m,\xi _{m-1}) \subseteq \varOmega$ combine into an *exponentially graded mesh* $\mathcal{T}$ that *does not* have locally bounded cardinality. In fact, $h_{\mathcal{T}} = h_{T_1} = 2^{-1}$ and $h_{\min ,\mathcal{T}} = h_{T_M} = 2^{1-M}$, which clearly contradicts the first requirement in Definition 2.4. Finally, note that an exponentially graded mesh can be interpreted as an algebraically graded mesh with grading exponent $\alpha = \infty$ (cf. Definition 3.4).

### Examples of local dual functions

In this subsection, we present a way to construct bases of $\mathbb {S}^{p,1}_{0}(\mathcal{T}\,\,)$ that is common in the *finite element* method. This scheme encompasses, in particular, the classical *hat functions* $\varphi _n \in \mathbb {S}^{1,1}_{0}(\mathcal{T}\,\,)$ as well as their generalization to $p \ge 1$ (Lagrange elements). Then, we show explicitly how to find a local dual system $\{\lambda _1,\dots ,\lambda _N\} \subseteq L^{2}(\varOmega )$ in the sense of Definition 2.6.

Let $p \ge 1$, $L {:=} \mathrm {dim}\,\mathbb {P}^{p}(\hat{T})$ and $N {:=} \mathrm {dim}\,\mathbb {S}^{p,1}_{0}(\mathcal{T})$. Let $\{\varphi _1,\dots ,\varphi _N\} \subseteq \mathbb {S}^{p,1}_{0}(\mathcal{T}\,\,)$ be a basis such that:

1) *Local supports:* For every $n \in \{1,\dots ,N\}$, there exists an element $T_n \in \mathcal{T}$ such that $T_n \in \mathrm {supp}_{\mathcal{T}}\,(\varphi _n) \subseteq \mathcal{T}(T_n)$.

2) *Simple structure:* There exists a basis of *shape functions* $\{\hat{\varphi }_1,\dots ,\hat{\varphi }_L\} \subseteq \mathbb {P}^{p}(\hat{T})$ that determines the shape of the basis elements. More precisely, for every $n \in \{1,\dots ,N\}$ and every $T \in \mathrm {supp}_{\mathcal{T}}\,(\varphi _n)$, there exists an index $\ell (n,T) \in \{1,\dots ,L\}$ such that $\varphi _n|_{T} = \hat{\varphi }_{\ell (n,T)} \circ F_T^{-1}$.

3) *Local distinctness:* The basis functions are *locally distinct* in the following sense: For all $n \ne m \in \{1,\dots ,N\}$ and all common $T \in \mathrm {supp}_{\mathcal{T}}\,(\varphi _n) \cap \mathrm {supp}_{\mathcal{T}}\,(\varphi _m)$, there holds $\ell (n,T) \ne \ell (m,T)$.

For each basis function $\varphi _n$ we fix an element $T_n \in \mathcal{T}$ as in 1). Note that a standard scaling argument $T \leftrightarrow \hat{T}$ readily provides the following relation:

$$\begin{aligned} \forall n \in \{1,\dots ,N\}: \quad \quad \Vert \varphi _n\Vert _{L^{2}(\varOmega )} \eqsim h_{T_n}^{d/2}. \end{aligned}$$

For the construction of the dual functions $\lambda _n \in L^{2}(\varOmega )$, let $\{\hat{\lambda }_1,\dots ,\hat{\lambda }_L\} \subseteq \mathbb {P}^{p}(\hat{T}\,\,)$ be the unique set of *dual shape functions*, i.e. $\langle \hat{\varphi }_{\ell },\hat{\lambda }_k\rangle _{L^{2}(\hat{T}\,\,)} = \delta _{\ell k}$ for all $\ell ,k \in \{1,\dots ,L\}$. We define the function $\lambda _n \in \mathbb {S}^{p,0}(\mathcal{T}\,\,) \subseteq L^{2}(\varOmega )$ in a piecewise manner: For every $T \ne T_n$, we set $\lambda _n|_{T} {:=} 0$, whereas

$$\begin{aligned} \lambda _n|_{T_n} {:=} \,|\mathrm {det}\,\nabla _{} F_{T_n}|^{-1} \,\cdot \,(\hat{\lambda }_{\ell (n,T_n)} \circ F_{T_n}^{-1}). \end{aligned}$$

#### Lemma 3.6

*The subset* $\{\lambda _1,\dots ,\lambda _N\} \subseteq L^{2}(\varOmega )$ *constitutes a local dual system in the sense of Definition* 2.6.

#### Proof

From the definition of $\lambda _n$, it is clear that $\mathrm {supp}_{\mathcal{T}}\,(\lambda _n) = \{T_n\} \subseteq \mathcal{T}(T_n)$. As for the duality, let $n,m \in \{1,\dots ,N\}$. If $T_m \notin \mathrm {supp}_{\mathcal{T}}\,(\varphi _n)$, we have $m \ne n$ and therefore $\langle \varphi _n,\lambda _m\rangle _{L^{2}(\varOmega )} = 0 = \delta _{nm}$. In the remaining case $T_m \in \mathrm {supp}_{\mathcal{T}}\,(\varphi _n)$ we get

$$\begin{aligned} \langle \varphi _n,\lambda _m\rangle _{L^{2}(\varOmega )} = \langle \varphi _n,\lambda _m\rangle _{L^{2}(T_m)} = \langle \hat{\varphi }_{\ell (n,T_m)},\hat{\lambda }_{\ell (m,T_m)}\rangle _{L^{2}(\hat{T}\,\,)} = \delta _{\ell (n,T_m) \ell (m,T_m)} = \delta _{nm}. \end{aligned}$$

We compute $|\mathrm {det}\,\nabla _{} F_{T_m}| = |\hat{T}|_{}^{-1/2} |T_m|_{}^{1/2}$. Recalling that $|T|_{} \eqsim h_{T}^d$ for every element *T* in a shape-regular mesh $\mathcal{T}$, we obtain for all $m \in \{1,\dots ,N\}$ the relation

$$\begin{aligned} \Vert \lambda _m\Vert _{L^{2}(\varOmega )}&= |\mathrm {det}\,\nabla _{} F_{T_m}|^{-1} \Vert \hat{\lambda }_{\ell (m,T_m)} \circ F_{T_m}^{-1}\Vert _{L^{2}(T_m)} \\&= |\hat{T}|_{}^{1/2} |T_m|_{}^{-1/2} \Vert \hat{\lambda }_{\ell (m,T_m)}\Vert _{L^{2}(\hat{T}\,\,)} \eqsim h_{T_m}^{-d/2}. \end{aligned}$$

Finally, for every $T \in \mathcal{T}$, we consider the indices $ms(T) {:=} \{m\,|\,T_m = T\}$. Due to the duality formula from above, the system $\{\lambda _1,\dots ,\lambda _N\} \subseteq \mathbb {S}^{p,0}(\mathcal{T}\,\,)$ is linearly independent. As a consequence, there must hold $\# ms(T) \lesssim 1$. For every $\varvec{x} \in \mathbb {R}^N$ and every $T \in \mathcal{T}$, we estimate

$$\begin{aligned} \bigg \Vert \sum _{m=1}^{N} \varvec{x}_m \lambda _m\bigg \Vert _{L^{2}(T)}^2&= \bigg \Vert \sum _{m \in ms(T)} \varvec{x}_m \lambda _m\bigg \Vert _{L^{2}(T)}^2\\&\le \bigg (\sum _{m \in ms(T)} \Vert \lambda _m\Vert _{L^{2}(\varOmega )}^2 \bigg )\bigg (\sum _{m \in ms(T)} \varvec{x}_m^2 \bigg )\lesssim h_{T}^{-d} \sum _{m \in ms(T)} \varvec{x}_m^2. \end{aligned}$$

Summing over all elements $T \in \mathcal{T}$ then gives the asserted global stability bound. This concludes the proof. $\square$

### A representation formula for the inverse system matrix

In this subsection, we develop a representation formula for $\varvec{A}^{-1}$ in terms of three linear operators: Recall that $\varvec{A}^{-1}$ represents the action of solving the discrete model problem, so there must be a fundamental connection to the *discrete solution operator* $S_{\mathcal{T}}: L^{2}(\varOmega ) \longrightarrow \mathbb {S}^{p,1}_{0}(\mathcal{T}\,\,)$. Additionally, we need a way to turn coefficient vectors $\varvec{f} \in \mathbb {R}^N$ into functions $f \in L^{2}(\varOmega )$ that can be plugged into $S_{\mathcal{T}}$. For this purpose, we can use the dual functions $\lambda _n \in L^{2}(\varOmega )$ from Definition 2.6 and the corresponding *coordinate mapping* $\Lambda : \mathbb {R}^N \longrightarrow L^{2}(\varOmega )$. Finally, the image $S_{{\mathcal{T}}}\, \Lambda\, {\varvec{f}} \in {\mathbb{S}}^{p,1}_{0}({\mathcal{T\;}})$ must be converted back to a vector in $\mathbb {R}^N$. A straightforward approach would be to use the inverse $\varPhi ^{-1}$ of the *coordinate mapping* $\varPhi : \mathbb {R}^N \longrightarrow \mathbb {S}^{p,1}_{0}(\mathcal{T}\,\,)$ associated with the basis functions $\varphi _n \in \mathbb {S}^{p,1}_{0}(\mathcal{T}\,\,)$. But, as it turns out, it is advantageous to use the Hilbert space transpose $\Lambda ^T: L^{2}(\varOmega ) \longrightarrow \mathbb {R}^N$ instead.

First, let us recall the following classical result:

#### Lemma 3.7

*The bilinear form* $a(\cdot ,\cdot )$ *from Definition* 2.1*is coercive and continuous*:

$$\begin{aligned} \forall u,v \in H^{1}_{0}(\varOmega ): \quad \quad \Vert u\Vert _{H^{1}(\varOmega )}^2 \lesssim a(u,u), \quad \quad |a(u,v)| \lesssim \Vert u\Vert _{H^{1}(\varOmega )} \Vert v\Vert _{H^{1}(\varOmega )}. \end{aligned}$$

The precise definition of the solution operator $S_{\mathcal{T}}$ is given in the following Definition 3.8 and the coordinate mappings $\varPhi$ and $\Lambda$ are defined in Definition 3.9.

#### Definition 3.8

Let $a: H^{1}_{0}(\varOmega ) \times H^{1}_{0}(\varOmega ) \longrightarrow \mathbb {R}$ be the bilinear form of Definition 2.1. For every $f \in L^{2}(\varOmega )$, denote by $S_{\mathcal{T}}\, f \in \mathbb {S}^{p,1}_{0}(\mathcal{T}\,\,)$ the unique function satisfying the variational equality

$$\begin{aligned} \forall v \in \mathbb {S}^{p,1}_{0}(\mathcal{T}\,\,): \quad \quad a(S_{\mathcal{T}} \,f,v) = \langle f,v\rangle _{L^{2}(\varOmega )}. \end{aligned}$$

The linear mapping $S_{\mathcal{T}}: L^{2}(\varOmega ) \longrightarrow \mathbb {S}^{p,1}_{0}(\mathcal{T}\,\,)$ is called *discrete solution operator*.

Recall from Sect. 2.4 that existence and uniqueness of $S_{\mathcal{T}}\, f$ are provided by the Lax-Milgram Lemma. Additionally, there holds the *a priori* bound $\Vert S_{\mathcal{T}}\, f\Vert _{H^{1}(\varOmega )} \lesssim \Vert f\Vert _{L^{2}(\varOmega )}$.

#### Definition 3.9

Let $\{\varphi _1,\dots ,\varphi _N\} \subseteq \mathbb {S}^{p,1}_{0}(\mathcal{T}\,\,)$ be a basis and $\{\lambda _1,\dots ,\lambda _N\} \subseteq L^{2}(\varOmega )$ be a dual system compliant with Definition 2.6. We denote the corresponding coordinate mappings by

$$\begin{aligned} \varPhi : \left\{ \begin{array}{ccc} \mathbb {R}^N &{} \longrightarrow &{} \mathbb {S}^{p,1}_{0}(\mathcal{T}\,\,) \\ \varvec{x} &{} \longmapsto &{} \sum _{n=1}^{N} \varvec{x}_n \varphi _n \end{array}\right. , \quad \quad \quad \Lambda : \left\{ \begin{array}{ccc} \mathbb {R}^N &{} \longrightarrow &{} L^{2}(\varOmega ) \\ \varvec{x} &{} \longmapsto &{} \sum _{n=1}^{N} \varvec{x}_n \lambda _n \end{array}\right. . \end{aligned}$$

We summarize the most important properties of $\varPhi$ and $\Lambda$ in the following lemma. As usual, we use the notation $\mathrm {supp}_{}(\varvec{x}) {:=} \{n \in \{1,\dots ,N\}\,|\,\varvec{x}_n \ne 0\}$ for the *support* of a vector $\varvec{x} \in \mathbb {R}^N$. Furthermore, recall from Definition 2.8 the notation $\mathcal{T}(I) \subseteq \mathcal{T}$ for all abstract matrix index sets $I \subseteq \{1,\dots ,N\}$.

#### Lemma 3.10

*The Hilbert space transpose of* $\Lambda$ *is given by the operator*

$$\begin{aligned} \Lambda ^T: \left\{ \begin{array}{ccc} L^{2}(\varOmega ) &{} \longrightarrow &{} \mathbb {R}^N \\ v &{} \longmapsto &{} (\langle v,\lambda _n\rangle _{L^{2}(\varOmega )})_{n=1}^{N} \end{array}\right. . \end{aligned}$$

*The restriction of* $\Lambda ^T$ *to the subspace* $\mathbb {S}^{p,1}_{0}(\mathcal{T}\,\,) \subseteq L^{2}(\varOmega )$ *coincides with the inverse mapping* $\varPhi ^{-1}$. *More precisely, for all* $\varvec{x}, \varvec{y} \in \mathbb {R}^N$ *and all* $v \in \mathbb {S}^{p,1}_{0}(\mathcal{T}\,\,)$, *there hold the duality/inversion formulae*

$$\begin{aligned} \langle \varPhi \varvec{x},\Lambda \varvec{y}\rangle _{L^{2}(\varOmega )} = \langle \varvec{x},\varvec{y}\rangle _{2}, \quad \quad \quad \Lambda ^T \varPhi \varvec{x} = \varvec{x}, \quad \quad \quad \varPhi \Lambda ^T v = v. \end{aligned}$$

*Both* $\Lambda$ *and* $\Lambda ^T$ *preserve locality: For all* $\varvec{x} \in \mathbb {R}^N$, $v \in L^{2}(\varOmega )$ *and* $I \subseteq \{1,\dots ,N\}$, *we have*

$$\begin{aligned} \mathrm {supp}_{\mathcal{T}}\,(\Lambda \varvec{x}) \subseteq \mathcal{T}(\mathrm {supp}_{}(\varvec{x})), \quad \quad \quad \Vert \Lambda ^T v\Vert _{\ell ^{2}(I)} \le \Vert \Lambda \Vert _{} \Vert v\Vert _{L^{2}(\mathcal{T}(I))}. \end{aligned}$$

#### Proof

The operator $\Lambda ^T$ is indeed the Hilbert space transpose of $\Lambda$: For all $v \in L^{2}(\varOmega )$ and $\varvec{x} \in \mathbb {R}^N$, we compute

$$\begin{aligned} \langle \Lambda ^T v,\varvec{x}\rangle _{2} = \sum _{n=1}^{N} \langle v,\lambda _n\rangle _{L^{2}(\varOmega )} \varvec{x}_n = \bigg \langle v,\sum _{n=1}^{N} \varvec{x}_n \lambda _n\bigg \rangle _{L^{2}(\varOmega )} = \langle v,\Lambda \varvec{x}\rangle _{L^{2}(\varOmega )}. \end{aligned}$$

The duality formula is a direct consequence of the duality property $\langle \varphi _n,\lambda _m\rangle _{L^{2}(\varOmega )} = \delta _{nm}$ from Definition 2.6: For all $\varvec{x}, \varvec{y} \in \mathbb {R}^N$, we have

$$\begin{aligned} \langle \varPhi \varvec{x},\Lambda \varvec{y}\rangle _{L^{2}(\varOmega )} = \sum _{n,m=1}^{N} \varvec{x}_n \varvec{y}_m \langle \varphi _n,\lambda _m\rangle _{L^{2}(\varOmega )} = \sum _{n=1}^{N} \varvec{x}_n \varvec{y}_n = \langle \varvec{x},\varvec{y}\rangle _{2}. \end{aligned}$$

From this, we immediately get the inversion formula $\Lambda ^T \varPhi \varvec{x} = \varvec{x}$ as well. On the other hand, for every $v \in \mathbb {S}^{p,1}_{0}(\mathcal{T}\,\,)$, there holds $\varPhi \Lambda ^T v = \varPhi \Lambda ^T \varPhi \varPhi ^{-1} v = \varPhi \varPhi ^{-1} v = v$.

Next, we turn our attention to the preservation of locality by $\Lambda$:

$$\begin{aligned} \forall \varvec{x} \in \mathbb {R}^N: \quad \quad \mathrm {supp}_{\mathcal{T}}\,(\Lambda \varvec{x})&= \mathrm {supp}_{\mathcal{T}}\bigg (\sum _{n \in \mathrm {supp}_{}(\varvec{x})} \varvec{x}_n \lambda _n\bigg ) \subseteq \bigcup _{n \in \mathrm {supp}_{}(\varvec{x})} \mathrm {supp}_{\mathcal{T}}(\lambda _n) \\&{\mathop {=}\limits ^{\text {Def.~2.8}}} \mathcal{T}(\mathrm {supp}_{}(\varvec{x})). \end{aligned}$$

Finally, let $v \in L^{2}(\varOmega )$ and $I \subseteq \{1,\dots ,N\}$. Let $\kappa _{}^{} \in L^{\infty }(\varOmega )$ be a (discontinuous) cut-off function with $\kappa _{}^{}|_{\mathcal{T}(I)} \equiv 1$ and $\kappa _{}^{}|_{\mathcal{T}\backslash \mathcal{T}(I)} \equiv 0$. Then,

$$\begin{aligned} \Vert \Lambda ^T v\Vert _{\ell ^{2}(I)} = \Vert \Lambda ^T(\kappa _{}^{} v)\Vert _{\ell ^{2}(I)} \le \Vert \Lambda ^T(\kappa _{}^{} v)\Vert _{2} \le \Vert \Lambda ^T\Vert _{} \Vert \kappa _{}^{} v\Vert _{L^{2}(\varOmega )} = \Vert \Lambda \Vert _{} \Vert v\Vert _{L^{2}(\mathcal{T}(I))}, \end{aligned}$$

which finishes the proof. $\square$

#### Lemma 3.11

*The system matrix* $\varvec{A} \in \mathbb {R}^{N \times N}$ *from Definition* 2.9, *the discrete solution operator* $S_{\mathcal{T}}: L^{2}(\varOmega ) \longrightarrow \mathbb {S}^{p,1}_{0}(\mathcal{T}\,\,)$ *from Definition* 3.8, *and the coordinate mapping* $\Lambda : \mathbb {R}^N \longrightarrow L^{2}(\varOmega )$ *from Definition* 3.9*are related via the representation formula*

$$\begin{aligned} \forall \varvec{f} \in \mathbb {R}^N: \quad \quad \varvec{A}^{-1} \varvec{f} = \Lambda ^T S_{\mathcal{T}}\, \Lambda\, \varvec{f}. \end{aligned}$$

#### Proof

First, we establish a relationship between $\varvec{A}$ and *a* by means of the coordinate mapping $\varPhi$:

$$\begin{aligned} \forall \varvec{x}, \varvec{y} \in \mathbb {R}^N: \quad \quad \langle \varvec{A} \varvec{x},\varvec{y}\rangle _{2} {\mathop {=}\limits ^{\text {Def.~2.9}}} \sum _{n,m=1}^{N} a(\varphi _n,\varphi _m) \varvec{x}_n \varvec{y}_m {\mathop {=}\limits ^{\text {Def.~3.9}}} a(\varPhi \varvec{x},\varPhi \varvec{y}). \end{aligned}$$

Now, using the duality and inversion formulae from Lemma 3.10, we get

$$\begin{aligned} \forall \varvec{f},\varvec{y} \in \mathbb {R}^N: \quad \langle \varvec{A} \Lambda ^T S_{\mathcal{T}}\, \Lambda\,\varvec{f},\varvec{y}\rangle _{2}&= a(\varPhi \Lambda ^T S_{\mathcal{T}} \,\Lambda \,\varvec{f},\varPhi \varvec{y}) = a(S_{\mathcal{T}}\, \Lambda\, \varvec{f},\varPhi \varvec{y}) \\&{\mathop {=}\limits ^{\text {Def.~3.8}}} \langle \Lambda\, \varvec{f},\varPhi \varvec{y}\rangle _{L^{2}(\varOmega )} = \langle \varvec{f},\Lambda ^T\varPhi \varvec{y}\rangle _{2} = \langle \varvec{f},\varvec{y}\rangle _{2}. \end{aligned}$$

This readily implies the stated representation formula. $\square$

### Reduction from matrix level to function level

In this subsection, we rephrase the original *matrix* approximation problem as a *function* approximation problem. This will remove the abstract matrix indices $I \subseteq \{1,\dots ,N\}$ in favor of element clusters $\mathcal{B}\subseteq \mathcal{T}$. The following lemma facilitates a reduction from the full matrix to the individual matrix blocks.

#### Lemma 3.12

*Let* $\mathbb {P}$ *be a sparse hierarchical block partition as in Definition* 2.10. *Then, there holds the estimate*

$$\begin{aligned} \forall \varvec{B} \in \mathbb {R}^{N \times N}: \quad \quad \Vert \varvec{B}\Vert _{2} \lesssim \ln (N) \,\cdot \,\max _{(I,J) \in \mathbb {P}} \Vert \varvec{B}|_{I \times J}\Vert _{2}. \end{aligned}$$

#### Proof

In [21, Lemma 6.5.8] (see also [8, 19]), the bound

$$\begin{aligned} \Vert \varvec{B}\Vert _{2} \le C_{\mathrm {sparse}}(\mathbb {T}_{N \times N}^{}) \mathrm {depth}(\mathbb {T}_{N}^{}) \max _{(I,J) \in \mathbb {P}} \Vert \varvec{B}|_{I \times J}\Vert _{2} \end{aligned}$$

was established. Inserting the bounds for the cluster tree depth and sparsity constant from Definition 2.10 immediately gives the desired result. $\square$

The following lemma is the main step in shifting the original problem from matrices to function spaces. Note that the representation formula for $\varvec{A}^{-1}$ from Lemma 3.11 plays a crucial role in its proof.

#### Lemma 3.13

*Let* $(I,J) \in \mathbb {P}_{\mathrm {adm}}$ *and* $V \subseteq L^{2}(\varOmega )$ *be a finite-dimensional subspace. Then, there exist matrices* $\varvec{X} \in \mathbb {R}^{I \times r}$ *and* $\varvec{Y} \in \mathbb {R}^{J \times r}$ *with* $r \le \mathrm {dim}\,V$ *such that there holds the error bound*

$$\begin{aligned} \Vert \varvec{A}^{-1}|_{I \times J} - \varvec{X} \varvec{Y}^T\Vert _{2} \le \Vert \Lambda \Vert _{}^2 \,\cdot \,\sup _{\begin{subarray}{c} f \in L^{2}(\varOmega ): \\ \mathrm {supp}_{\mathcal{T}}\,(f) \subseteq \mathcal{T}(J) \end{subarray}} \inf _{v \in V}\; \frac{\Vert S_{\mathcal{T}}\, f - v\Vert _{L^{2}(\mathcal{T}(I))}}{\Vert f\Vert _{L^{2}(\varOmega )}}. \end{aligned}$$

#### Proof

We use the transposed coordinate mapping $\Lambda ^T: L^{2}(\varOmega ) \longrightarrow \mathbb {R}^N$ from Lemma 3.10 to define $\varvec{V} {:=} (\Lambda ^T V)|_{I} \subseteq \mathbb {R}^I$. Note that $r {:=} \mathrm {dim}\,\varvec{V} \le \mathrm {dim}\,V$. Next, let the columns of the matrix $\varvec{X} \in \mathbb {R}^{I \times r}$ be an $\ell ^{2}(I)$-orthonormal basis of $\varvec{V}$. In particular, the product $\varvec{X}\varvec{X}^T \in \mathbb {R}^{I \times I}$ represents the $\ell ^{2}(I)$-orthogonal projection from $\mathbb {R}^I$ onto $\varvec{V}$. Finally, set $\varvec{Y} {:=} (\varvec{A}^{-1}|_{I \times J})^T \varvec{X} \in \mathbb {R}^{J \times r}$.

For every $\varvec{f} \in \mathbb {R}^N$ with $\mathrm {supp}_{}(\varvec{f}) \subseteq J$, we get the bound

$$\begin{aligned}&\Vert (\varvec{A}^{-1}|_{I \times J} - \varvec{X} \varvec{Y}^T) \varvec{f}|_{J}\Vert _{\ell ^{2}(I)} = \Vert (\varvec{I} - \varvec{X} \varvec{X}^T) (\varvec{A}^{-1} \varvec{f})|_{I}\Vert _{\ell ^{2}(I)} = \inf _{\varvec{v} \in \varvec{V}} \Vert (\varvec{A}^{-1} \varvec{f})|_{I} - \varvec{v}\Vert _{\ell ^{2}(I)} \\&\quad {\mathop {=}\limits ^{\text {Lem.~3.11}}} \inf _{v \in V} \Vert \Lambda ^T(S_{\mathcal{T}}\, \Lambda\, \varvec{f} - v)\Vert _{\ell ^{2}(I)} {\mathop {\le }\limits ^{\text {Lem.~3.10}}} \Vert \Lambda \Vert _{} \,\cdot \,\inf _{v \in V} \Vert S_{\mathcal{T}}\, \Lambda\, \varvec{f} - v\Vert _{L^{2}(\mathcal{T}(I))}. \end{aligned}$$

We can divide both sides by $\Vert \varvec{f}\Vert _{\ell ^{2}(J)}$, take suprema and substitute $f {:=} \Lambda \varvec{f} \in L^{2}(\varOmega )$. Finally, we use $\mathrm {supp}_{\mathcal{T}}\,(f) = \mathrm {supp}_{\mathcal{T}}\,(\Lambda\, \varvec{f}) \subseteq \mathcal{T}(\mathrm {supp}_{}(\varvec{f})) \subseteq \mathcal{T}(J)$ and $\Vert \varvec{f}\Vert _{\ell ^{2}(J)}^{-1} \le \Vert \Lambda \Vert _{} \Vert f\Vert _{L^{2}(\varOmega )}^{-1}$ to get the desired result. $\square$

A thorough understanding of the preceding lemma is absolutely fundamental for the subsequent sections. Therefore, let us recall its interpretation from Sect. 3.1:

Given $\mathcal{B}$, $\mathcal{D}\subseteq \mathcal{T}$ with $0 < \mathrm {diam}_{\mathcal{T}}\,(\mathcal{B}) \le \sigma _{\mathrm {adm}}\mathrm {dist}_{\mathcal{T}}\,(\mathcal{B},\mathcal{D})$ and $L \in \mathbb {N}$, how can we construct a subspace $V_{\mathcal{B},\mathcal{D},L} \subseteq L^{2}(\varOmega )$ of dimension $\mathrm {dim}\,V_{\mathcal{B},\mathcal{D},L} \lesssim L^{\kappa }$ (for some fixed $\kappa \ge 1$) that satisfies the error bound

$$\begin{aligned} \inf _{v \in V_{\mathcal{B},\mathcal{D},L}} \Vert S_{\mathcal{T}}\, f - v\Vert _{L^{2}(\mathcal{B})} \lesssim 2^{-L} \Vert f\Vert _{L^{2}(\mathcal{D})}, \end{aligned}$$

for all source functions $f \in L^{2}(\varOmega )$ with $\mathrm {supp}_{\mathcal{T}}\,(f) \subseteq \mathcal{D}$?

### The discrete cut-off operator

The notion of *cluster inflation* provides a means of enlarging a given cluster by a predefined threshold with respect to the mesh metric $\mathrm {dist}_{\mathcal{T}}\,(\cdot ,\cdot )$ from Definition 2.3. This is one of the core concepts in our proof and will be used extensively. We acknowledge this fact with tight notation:

#### Definition 3.14

For every cluster $\mathcal{B}\subseteq \mathcal{T}$ and every radius $\delta \ge 0$, we introduce the *inflated cluster*

$$\begin{aligned} {\mathcal{B}}^{\delta }_{} {:=} \{T \in \mathcal{T}\,|\,\mathrm {dist}_{\mathcal{T}}\,(T,\mathcal{B}) \le \delta \}. \end{aligned}$$

We summarize the most important facts about the mesh metric and inflated clusters in the subsequent lemma.

#### Lemma 3.15

*The mesh metric* $\mathrm {dist}_{\mathcal{T}}\,(\cdot ,\cdot )$ *from Definition* 2.3*defines a metric on* $\mathcal{T}$. *There holds the triangle type inequality*

$$\begin{aligned} \forall \mathcal{A},\mathcal{B},\mathcal{C}\subseteq \mathcal{T}: \quad \quad \mathrm {dist}_{\mathcal{T}}\,(\mathcal{A},\mathcal{C}) \le \mathrm {dist}_{\mathcal{T}}\,(\mathcal{A},\mathcal{B}) + \mathrm {diam}_{\mathcal{T}}\,(\mathcal{B}) + \mathrm {dist}_{\mathcal{T}}\,(\mathcal{B},\mathcal{C}). \end{aligned}$$

*For every element* $T \in \mathcal{T}$ *and every neighbor* $S \in \mathcal{T}(T)$, [*cf.* (2.1)] *the distance is bounded by* $\mathrm {dist}_{\mathcal{T}}\,(T,S) \le \sigma _{\mathrm {shp}}h_{T}$. *On the other hand, for every* $S \in \mathcal{T}\,\,\backslash \{T\}$, *we have the lower bound* $\mathrm {dist}_{\mathcal{T}}\,(T,S) \ge \sigma _{\mathrm {shp}}^{-1}(h_{T} + h_{S})$. *Additionally, for every cluster* $\mathcal{B}\subseteq \mathcal{T}$, *there holds* $h_{\mathcal{B}} \le \max \{h_{\min ,\mathcal{B}}, \sigma _{\mathrm {shp}}\mathrm {diam}_{\mathcal{T}}\,(\mathcal{B})\}$.

*When dealing with a second mesh* $\mathcal{S}\subseteq \mathrm {Pow}\,(\varOmega )$, *cluster diameters are essentially equivalent*:

$$\begin{aligned} \forall \mathcal{B}\subseteq \mathcal{T}: \quad \quad \mathrm {diam}_{\mathcal{S}}(\mathcal{S}\,(\bigcup \mathcal{B})) \le \mathrm {diam}_{\mathcal{T}}\,(\mathcal{B}) + 2h_{\mathcal{B}} + 2h_{\mathcal{S}(\bigcup \mathcal{B})}. \end{aligned}$$

*Finally, consider clusters* $\mathcal{B}\subseteq \mathcal{C}\subseteq \mathcal{T}$ *and inflation radii* $\delta$, $\varepsilon \ge 0$. *Then*, $\mathcal{B}\subseteq {\mathcal{B}}^{\delta }_{} \subseteq ({\mathcal{B}}^{\delta }_{})^{\varepsilon }_{} \subseteq {\mathcal{B}}^{\delta +\varepsilon }_{} \subseteq {\mathcal{C}}^{\delta +\varepsilon }_{}$. *For the cluster patch* $\mathcal{T}(\mathcal{B})$ *we have the inclusion* $\mathcal{T}(\mathcal{B}) \subseteq {\mathcal{B}}^{\sigma _{\mathrm {shp}}h_{\mathcal{B}}}_{}$. *Finally*, $\mathrm {diam}_{\mathcal{T}}\,({\mathcal{B}}^{\delta }_{}) \le \mathrm {diam}_{\mathcal{T}}\,(\mathcal{B}) + 2\delta$ *and* $h_{{\mathcal{B}}^{\delta }_{}} \le \max \{h_{\mathcal{B}}, \sigma _{\mathrm {shp}}\delta \}$.

#### Proof

Both the verification of the metric axioms for $\mathrm {dist}_{\mathcal{T}}(\cdot ,\cdot )$ and the triangle type inequality are straightforward.

Let $T \in \mathcal{T}$ and $S \in \mathcal{T}(T)$. From the assumption on shape regularity following Definition 2.2, we know that $x_S \in S \subseteq \bigcup \mathcal{T}(T) \subseteq \mathrm {Ball}_{2}(x_T,\sigma _{\mathrm {shp}}h_{T}\,\,)$. This yields $\mathrm {dist}_{\mathcal{T}}(T,S) = \Vert x_S-x_T\Vert _{2} \le \sigma _{\mathrm {shp}}h_{T}$. Next, let $T \in \mathcal{T}$ and $S \in \mathcal{T}\,\,\backslash \{T\}$. Again, from shape regularity, we know that $\mathrm {Ball}_{2}(x_S,\sigma _{\mathrm {shp}}^{-1}h_{S}) \subseteq S$ and $\mathrm {Ball}_{2}(x_T,\sigma _{\mathrm {shp}}^{-1}h_{T}\,\,) \subseteq T$. Since *S* and *T* are disjoint, the inscribed balls have to be disjoint, too. We conclude $\mathrm {dist}_{\mathcal{T}}(T,S) = \Vert x_S-x_T\Vert _{2} \ge \sigma _{\mathrm {shp}}^{-1}(h_{T}+h_{S})$.

Next, let $\mathcal{B}\subseteq \mathcal{T}$. In the case $\# \mathcal{B} = 1$ we clearly have $h_{\mathcal{B}} = h_{\min ,\mathcal{B}}$. In the case $\# \mathcal{B} \ge 2$ we can choose distinct elements $B \ne B_{\max } \in \mathcal{B}$ with $h_{B_{\max }} = h_{\mathcal{B}}$ and conclude $h_{\mathcal{B}} = h_{B_{\max }} \le \sigma _{\mathrm {shp}}\mathrm {dist}_{\mathcal{T}}\,(B,B_{\max }) \le \sigma _{\mathrm {shp}}\mathrm {diam}_{\mathcal{T}}\,(\mathcal{B})$.

Let $\mathcal{S}\subseteq \mathrm {Pow}(\varOmega )$ be an additional mesh, $\mathcal{B}\subseteq \mathcal{T}$ and $B {:=} \bigcup \mathcal{B}\subseteq \mathbb {R}^d$ the associated domain. For every $S \in \mathcal{S}(B)$ there exists a $T(S) \in \mathcal{B}$ with $\overline{T(S)} \cap \overline{S} \ne \emptyset$. Clearly, $\Vert x_S-x_{T(S)}\Vert _{2} \le h_{S} + h_{T(S)}$. We conclude

$$\begin{aligned} \mathrm {diam}_{\mathcal{S}}(\mathcal{S}(B))&\le \max _{R,S \in \mathcal{S}(B)} \Vert x_S-x_{T(S)}\Vert _{2} + \Vert x_{T(S)}-x_{T(R)}\Vert _{2} + \Vert x_{T(R)}-x_R\Vert _{2} \\&\le \mathrm {diam}_{\mathcal{T}}(\mathcal{B}) + 2h_{\mathcal{B}} + 2h_{\mathcal{S}(B)}. \end{aligned}$$

The inclusion chain for inflated clusters follows directly from Definition 3.14 and the triangle type inequality above.

To see the inclusion of a cluster patch in some inflated cluster, let $\mathcal{B}\subseteq \mathcal{T}$. Then, for every $T \in \mathcal{T}(\mathcal{B})$ there exists a $B \in \mathcal{B}$ with $T \in \mathcal{T}(B)$. We get $\mathrm {dist}_{\mathcal{T}}\,(T,\mathcal{B}) \le \mathrm {dist}_{\mathcal{T}}\,(T,B) \le \sigma _{\mathrm {shp}}h_{B} \le \sigma _{\mathrm {shp}}h_{\mathcal{B}}$, i.e., the inclusion $\mathcal{T}(\mathcal{B}) \subseteq {\mathcal{B}}^{\sigma _{\mathrm {shp}}h_{\mathcal{B}}}_{}$.

Once again, let $\mathcal{B}\subseteq \mathcal{T}$ and $\delta \ge 0$. The inequality $\mathrm {diam}_{\mathcal{T}}\,({\mathcal{B}}^{\delta }_{}) \le \mathrm {diam}_{\mathcal{T}}\,(\mathcal{B}) + 2\delta$ can be derived from the mesh metric’s triangle inequality. Finally, to find an upper bound for $h_{{\mathcal{B}}^{\delta }_{}}$, consider an arbitrary $T \in {\mathcal{B}}^{\delta }_{}$. By definition, there exists a $B \in \mathcal{B}$ with $\mathrm {dist}_{\mathcal{T}}\,(T,B) \le \delta$. In the case $T=B$ we get $h_{T} = h_{B} \le h_{\mathcal{B}}$ and in the remaining case $T \ne B$ we have $h_{T} \le \sigma _{\mathrm {shp}}\mathrm {dist}_{\mathcal{T}}\,(T,B) \le \sigma _{\mathrm {shp}}\delta$. $\square$

For the construction of the cut-off function $\kappa _{\mathcal{B}}^{\delta }$ in Lemma 3.18 we will use a variant of the classical *Clément operator*, [10].

#### Definition 3.16

Let $\mathcal{N}\subseteq \overline{\varOmega }$ be the nodes of the mesh $\mathcal{T}$ and denote by $\{b_N\,|\,N \in \mathcal{N}\} \subseteq \mathbb {S}^{1,1}(\mathcal{T}\,\,)$ the well-known *hat-functions*, i.e., $b_N(M) = \delta _{NM}$. We write $\langle v\rangle _{T} {:=} |T|_{}^{-1} \int \displaylimits _{T}^{} v \,\mathrm {d}x \in \mathbb {R}$ for the mean value of a function $v \in L^{2}(\varOmega )$ on an element $T \in \mathcal{T}$. Now, the *Clément operator* $J_{\mathcal{T}}: L^{2}(\varOmega ) \longrightarrow \mathbb {S}^{1,1}(\mathcal{T}\,\,)$ is defined in a nodewise fashion: For every $v \in L^{2}(\varOmega )$, we set $J_{\mathcal{T}} v {:=} \sum _{N \in \mathcal{N}} \beta _N b_N$, where the nodal value $\beta _N$ is given by

$$\begin{aligned} \beta _N {:=} \frac{1}{\# \mathcal{T}(N)} \sum _{T \in \mathcal{T}(N)} \langle v\rangle _{T}. \end{aligned}$$

#### Lemma 3.17

*The linear operator* $J_{\mathcal{T}}$ *has a local projection property*: *Given a cluster* $\mathcal{B}\subseteq \mathcal{T}$ *and a function* $v \in L^{2}(\varOmega )$ *with* $v|_{\mathcal{T}(\mathcal{B})} \equiv \mathrm {const}$, *there holds* $(J_{\mathcal{T}} v)|_{\mathcal{B}} = v|_{\mathcal{B}}$. *Furthermore*, $J_{\mathcal{T}}$ *preserves discrete supports: For every* $q \ge 0$ *and every* $v \in \mathbb {S}^{q,0}(\mathcal{T}\,\,)$, *there holds* $\mathrm {supp}_{\mathcal{T}}\,(J_{\mathcal{T}} v) \subseteq \mathcal{T}(\mathrm {supp}_{}(v))$. *Moreover*, $J_{\mathcal{T}}$ *preserves ranges: For every* $v \in \mathbb {S}^{1,0}(\mathcal{T}\,\,)$ *with* $0 \le v \le 1$ *there also holds* $0 \le J_{\mathcal{T}} v \le 1$. *Finally, we have the stability bound*

$$\begin{aligned} \forall v \in L^{2}(\varOmega ): \forall T \in \mathcal{T}: \quad \quad h_{T} |J_{\mathcal{T}} v|_{W^{1,\infty }(T)} \lesssim \max _{S \in \mathcal{T}(T)} |\langle v\rangle _{T} - \langle v\rangle _{S}|. \end{aligned}$$

#### Proof

We only show the stability bound: For every $w \in \mathbb {S}^{1,0}(\mathcal{T}\,\,)$ and every $T \in \mathcal{T}$, the inverse inequality from Lemma 3.20 provides the estimate $h_{T} |w|_{W^{1,\infty }(T)} \lesssim \Vert w\Vert _{L^{\infty }(T)} = \max _{N \in \mathcal{N}(T)} |w(N)|$, where $\mathcal{N}(T)$ denotes the nodes of the element *T*. Since $|\cdot |_{W^{1,\infty }(T)}$ annihilates constants, we also get $h_{T} |w|_{W^{1,\infty }(T)} \lesssim \max _{N,M \in \mathcal{N}(T)} |w(N)-w(M)|$. Inserting $w {:=} J_{\mathcal{T}} v \in \mathbb {S}^{1,1}(\mathcal{T}\,\,)$ and using $(J_{\mathcal{T}} v)(N) = \beta _N$, the asserted stability bound follows readily. $\square$

The discretized model problem $a(u,v) = \langle f,v\rangle _{L^{2}(\varOmega )}$ was phrased in terms of *global* functions $u,v \in \mathbb {S}^{p,1}_{0}(\mathcal{T}\,\,)$. But if we plug in a function *v* with local support, e.g., $\mathrm {supp}_{\mathcal{T}}(v) \subseteq \mathcal{B}$ for some prescribed cluster $\mathcal{B}\subseteq \mathcal{T}$, we can extract local information about *u* on $\mathcal{B}$. This motivates the usage of *discrete cut-off functions*.

#### Lemma 3.18

*Let* $\mathcal{B}\subseteq \mathcal{T}$ *and* $\delta >0$ *with* $4\sigma _{\mathrm {shp}}^3 h_{\mathcal{B}} \le \delta \lesssim 1$. *Then, there exists a* *discrete cut-off function* $\kappa _{\mathcal{B}}^{\delta }$ *with*

$$\begin{aligned} \kappa _{\mathcal{B}}^{\delta } \in \mathbb {S}^{1,1}(\mathcal{T}\,\,), \ \quad \mathrm {supp}_{\mathcal{T}}\,(\kappa _{\mathcal{B}}^{\delta }) \subseteq {\mathcal{B}}^{\delta }_{}, \ \quad \kappa _{\mathcal{B}}^{\delta }|_{\mathcal{B}} \equiv 1, \ \quad 0 \le \kappa _{\mathcal{B}}^{\delta } \le 1, \ \quad \Vert \kappa _{\mathcal{B}}^{\delta }\Vert _{W^{1,\infty }(\varOmega )} \lesssim \frac{1}{\delta }. \end{aligned}$$

#### Proof

We abbreviate $\varepsilon {:=} \delta /(4\sigma _{\mathrm {shp}}^2) > 0$ and consider a step function $\kappa _{}^{} \in \mathbb {S}^{0,0}(\mathcal{T}\,\,)$ defined by

$$\begin{aligned} \forall T \in \mathcal{T}: \quad \quad \kappa _{}^{}|_{T} {:=} \max \{0, 1-\mathrm {dist}_{\mathcal{T}}\,(T,\mathcal{T}(\mathcal{B}))/\varepsilon \} \in \mathbb {R}. \end{aligned}$$

From the definition we immediately get $\mathrm {supp}_{\mathcal{T}}(\kappa _{}^{}) \subseteq {\mathcal{T}(\mathcal{B})}^{\varepsilon }_{}$ and $\kappa _{}^{}|_{\mathcal{T}(\mathcal{B})} \equiv 1$ as well as $0 \le \kappa _{}^{} \le 1$. (Recall that $\mathcal{T}(\mathcal{B})$ are all patch elements of $\mathcal{B}$ and ${\mathcal{T}(\mathcal{B})}^{\varepsilon }_{}$ is the corresponding inflated cluster by a radius of $\varepsilon$.) Next, for every $T \in \mathcal{T}$ and every neighbor $S \in \mathcal{T}(T)$, we apply the triangle inequality from Lemma 3.15 to the clusters $\{T\}, \{S\}, \mathcal{T}(\mathcal{B})$ and derive $\mathrm {dist}_{\mathcal{T}}\,(T,\mathcal{T}(\mathcal{B})) \le \mathrm {dist}_{\mathcal{T}}\,(T,S) + \mathrm {dist}_{\mathcal{T}}\,(S,\mathcal{T}(\mathcal{B}))$. (Recall from Definition 2.3 that $\mathrm {diam}_{\mathcal{T}}\,(S) = 0$, since $\{S\}$ contains only one element.) Exploiting the Lipschitz continuity of $t \mapsto \max \{0,t\}$, we get the error bound

$$\begin{aligned} |\kappa _{}^{}|_{T} - \kappa _{}^{}|_{S}| \le \frac{|\mathrm {dist}_{\mathcal{T}}\,(T,\mathcal{T}(\mathcal{B})) - \mathrm {dist}_{\mathcal{T}}\,(S,\mathcal{T}(\mathcal{B}))|}{\varepsilon } {\mathop {\le }\limits ^{}} \frac{\mathrm {dist}_{\mathcal{T}}\,(T,S)}{\varepsilon } {\mathop {\lesssim }\limits ^{\text {Lem.~3.15}}} \frac{h_{T}}{\varepsilon } \eqsim \frac{h_{T}}{\delta }. \end{aligned}$$

We use the Clément operator $J_{\mathcal{T}}: L^{2}(\varOmega ) \longrightarrow \mathbb {S}^{1,1}(\mathcal{T}\,\,)$ from Definition 3.16 to define $\kappa _{\mathcal{B}}^{\delta } {:=} J_{\mathcal{T}} \kappa _{}^{} \in \mathbb {S}^{1,1}(\mathcal{T}\,\,)$. For the support of $\kappa _{\mathcal{B}}^{\delta }$ we compute

$$\begin{aligned} \mathrm {supp}_{\mathcal{T}}\,(\kappa _{\mathcal{B}}^{\delta })&{\mathop {\subseteq }\limits ^{\text {Lem.~3.17}}} \mathcal{T}(\mathrm {supp}_{\mathcal{T}}\,(\kappa _{}^{})) \subseteq \mathcal{T}({\mathcal{T}(\mathcal{B})}^{\varepsilon }_{}) {\mathop {\subseteq }\limits ^{\text {Lem.~3.15}}} {\mathcal{B}}^{(1+\sigma _{\mathrm {shp}}^2)(\sigma _{\mathrm {shp}}h_{\mathcal{B}} + \varepsilon )}_{} \\&\subseteq {\mathcal{B}}^{2\sigma _{\mathrm {shp}}^3 h_{\mathcal{B}} + \delta /2}_{} \subseteq {\mathcal{B}}^{\delta }_{}, \end{aligned}$$

where in the last step we used $\delta \ge 4\sigma _{\mathrm {shp}}^3 h_{\mathcal{B}}$.

From Lemma 3.17 and $\kappa _{}^{}|_{\mathcal{T}(\mathcal{B})} \equiv 1$ we get $\kappa _{\mathcal{B}}^{\delta }|_{\mathcal{B}} \equiv 1$. Moreover, $0 \le \kappa _{}^{} \le 1$ yields $0 \le \kappa _{\mathcal{B}}^{\delta } \le 1$. This implies, in particular, $\Vert \kappa _{\mathcal{B}}^{\delta }\Vert _{L^{\infty }(\varOmega )} \le 1 \lesssim \delta ^{-1}$, where we used the assumption $\delta \lesssim 1$. The remaining bound $|\kappa _{\mathcal{B}}^{\delta }|_{W^{1,\infty }(\varOmega )} \lesssim \delta ^{-1}$ follows from

$$\begin{aligned} \forall T \in \mathcal{T}: \quad \quad h_{T} |\kappa _{\mathcal{B}}^{\delta }|_{W^{1,\infty }(T)} {\mathop {\lesssim }\limits ^{\text {Lem.~3.17}}} \max _{S \in \mathcal{T}(T)} |\kappa _{}^{}|_{T} - \kappa _{}^{}|_{S}| \lesssim \frac{h_{T}}{\delta }. \end{aligned}$$

This finishes the proof. $\square$

Given a cluster $\mathcal{B}\subseteq \mathcal{T}$ and a distance $\delta >0$, the discrete cut-off function $\kappa _{\mathcal{B}}^{\delta }$ allows us to “restrict” a function $v \in \mathbb {S}^{p,1}(\mathcal{T}\,\,)$ to the subdomain $\bigcup {\mathcal{B}}^{\delta }_{} \subseteq \varOmega$ while preserving continuity. This can be achieved by simply multiplying *v* with $\kappa _{\mathcal{B}}^{\delta }$. Note that the product $\kappa _{\mathcal{B}}^{\delta } v$ has polynomial degree $p+1$, rather than *p*. To mitigate this drawback, we can simply re-interpolate the result with an operator of order *p*.

#### Definition 3.19

Let $p \ge 1$ and denote by $\hat{I}^p: C^{0}(\overline{\hat{T}}) \longrightarrow \mathbb {P}^{p}(\hat{T})$ the *(local) Lagrange interpolation operator* on the reference element $\hat{T}$. The *(global) Lagrange interpolation operator* $I_{\mathcal{T}}^p: C^{0}_{\mathrm {pw}}(\mathcal{T}\,\,) \longrightarrow \mathbb {S}^{p,0}(\mathcal{T}\,\,)$ is defined in a piecewise manner: For every $v \in C^{0}_{\mathrm {pw}}(\mathcal{T}\,\,)$ and every $T \in \mathcal{T}$, we set

$$\begin{aligned} (I_{\mathcal{T}}^p v)|_{T} {:=} \,\hat{I}^p(v \circ F_T) \circ F_T^{-1}. \end{aligned}$$

In order to derive a useful stability estimate for $I_{\mathcal{T}}^p$, we use a standard elementwise inverse inequality, which follows from scaling arguments.

#### Lemma 3.20

*Let* $k,\ell \in \mathbb {N}_0$ *with* $k \ge \ell \ge 0$, $q \in [1,\infty ]$ *and* $p \ge 0$. *Then, for all discrete functions* $v \in \mathbb {S}^{p,0}(\mathcal{T}\,\,)$ *and all elements* $T \in \mathcal{T}$, *there holds the* *inverse inequality*

$$\begin{aligned} h_{T}^k |v|_{W^{k,q}(T)} \lesssim h_{T}^{\ell } |v|_{W^{\ell ,q}(T)}. \end{aligned}$$

The properties of the Lagrange interpolation operator $I_{\mathcal{T}}^p$ are very similar to those of the Clément operator $J_{\mathcal{T}}$ from Definition 3.16. For the sake of completeness, we include them in the following lemma.

#### Lemma 3.21

*Let* $p \ge 1$. *The linear operator* $I_{\mathcal{T}}^p$ *has a local projection property: Given a cluster* $\mathcal{B}\subseteq \mathcal{T}$ *and a function* $v \in C^{0}_{\mathrm {pw}}(\mathcal{T}\,\,)$ *with* $v \in \mathbb {S}^{p,0}(\mathcal{B})$, *there holds* $(I_{\mathcal{T}}^p v)|_{\mathcal{B}} = v|_{\mathcal{B}}$. *Furthermore*, $I_{\mathcal{T}}^p$ *preserves global continuity and homogeneous* *boundary values: For every* $v \in C^{0}(\overline{\varOmega })$, *there* *holds* $I_{\mathcal{T}}^p v \in \mathbb {S}^{p,1}(\mathcal{T}\,\,)$. *Similarly, if* $v \in C^{0}(\overline{\varOmega })$ *with* $v|_{\partial \varOmega } \equiv 0$, *then* $I_{\mathcal{T}}^p v \in \mathbb {S}^{p,1}_{0}(\mathcal{T}\,\,)$. *Moreover*, $I_{\mathcal{T}}^p$ *preserves discrete supports: For every* $q \ge 0$ *and every* $v \in \mathbb {S}^{q,0}(\mathcal{T}\,\,)$, *we have* $\mathrm {supp}_{\mathcal{T}}\,(I_{\mathcal{T}}^p v) \subseteq \mathrm {supp}_{\mathcal{T}}\,(v)$. *Finally, for all* $q \ge 0$, $v \in \mathbb {S}^{q,0}(\mathcal{T}\,\,)$ *and* $T \in \mathcal{T}$, *there hold the following stability and error estimates* (*with constants depending on* *q*):

$$\begin{aligned} \begin{array}{lrcl} \forall m \in \{0,\dots ,p+1\}: \quad &{} |I_{\mathcal{T}}^p v|_{H^{m}(T)} &{}\lesssim &{} |v|_{H^{m}(T)}, \\ &{} \sum _{\ell =0}^{p+1} h_{T}^{\ell } |(\mathrm {id}- I_{\mathcal{T}}^p)(v)|_{H^{\ell }(T)} &{}\lesssim &{} h_{T}^{p+1} |v|_{H^{p+1}(T)}. \end{array} \end{aligned}$$

#### Proof

We briefly sketch the proof of the stability and error bounds: The mapping $v \mapsto \Vert \hat{I}^p v\Vert _{L^{2}(\hat{T}\,\,)} + |v|_{H^{p+1}(\hat{T}\,\,)}$ defines a norm on the finite-dimensional space $\mathbb {P}^{q}(\hat{T}\,\,)$. Hence, by norm equivalence, $\Vert v\Vert _{H^{p+1}(\hat{T}\,\,)} \lesssim \Vert \hat{I}^p v\Vert _{L^{2}(\hat{T}\,\,)} + |v|_{H^{p+1}(\hat{T}\,\,)}$ for all $v \in \mathbb {P}^{q}(\hat{T}\,\,)$. Inserting $v {:=} w-\hat{I}^p w$ for arbitrary $w \in \mathbb {P}^{q}(\hat{T}\,\,)$ results in the bound $\Vert w-\hat{I}^p w\Vert _{H^{p+1}(\hat{T}\,\,)} \lesssim |w|_{H^{p+1}(\hat{T}\,\,)}$. Finally, a standard scaling argument $\hat{T}\leftrightarrow T$ yields the desired error estimate on *T*. As for the stability bound, we perform a straightforward triangle inequality on *T*, reuse the already proven error bound and finish off with the inverse inequality from Lemma 3.20. $\square$

#### Remark 3.22

The fact that $I_{\mathcal{T}}^p$ preserves global continuity and homogeneous boundary values hinges on an implicit assumption about the (local) interpolation points used by the local Lagrange interpolation operator $\hat{I}^p$. Recall from Definition 2.2 that the reference element $\hat{T}\subseteq \mathbb {R}^d$ is a simplex and thus delimited by $d+1$ hyperplanes. The interpolation points on each hyperplane $\hat{E}$ must be unisolvent for the space $\mathbb {P}^{p}(\hat{E})$. Then, in particular, every polynomial $v \in \mathbb {P}^{p}(\hat{T}\,\,)$ vanishing at the interpolation points in $\hat{E}$ must already vanish everywhere on $\hat{E}$. This property readily implies that homogeneous boundary values are preserved by the global operator $I_{\mathcal{T}}^p$. Finally, the distribution of interpolation points on each hyperplane $\hat{E}$ must be “symmetric”. More precisely, if two elements $T_1$, $T_2 \in \mathcal{T}$ share a common hyperplane, we require the corresponding interpolation points to align perfectly. In this case, using the same argument as before, the operator $I_{\mathcal{T}}^p$ preserves global continuity indeed.

As our next step, we encapsulate the aforementioned “cut-off” process in a linear operator.

#### Definition 3.23

Let $\mathcal{B}\subseteq \mathcal{T}$ and $\delta >0$ with $4\sigma _{\mathrm {shp}}^3 h_{\mathcal{B}} \le \delta \lesssim 1$ and denote by $\kappa _{\mathcal{B}}^{\delta } \in \mathbb {S}^{1,1}(\mathcal{T}\,\,)$ the discrete cut-off function from Lemma 3.18. Furthermore, denote by $I_{\mathcal{T}}^p: C^{0}_{\mathrm {pw}}(\mathcal{T}\,\,) \longrightarrow \mathbb {S}^{p,0}(\mathcal{T}\,\,)$ the Lagrange interpolation operator from Definition 3.19. We define the *discrete cut-off operator*

$$\begin{aligned} K_{\mathcal{B}}^{\delta }: \left\{ \begin{array}{ccc} \mathbb {S}^{p,1}(\mathcal{T}\,\,) &{} \longrightarrow &{} \mathbb {S}^{p,1}(\mathcal{T}\,\,) \\ v &{} \longmapsto &{} I_{\mathcal{T}}^p(\kappa _{\mathcal{B}}^{\delta } v) \end{array}\right. . \end{aligned}$$

The discrete cut-off operator $K_{\mathcal{B}}^{\delta }$ inherits its core properties from $I_{\mathcal{T}}^p$.

#### Lemma 3.24

*Let* $\mathcal{B}\subseteq \mathcal{T}$ *and* $\delta >0$ *with* $4\sigma _{\mathrm {shp}}^3 h_{\mathcal{B}} \le \delta \lesssim 1$. *For all* $v \in \mathbb {S}^{p,1}(\mathcal{T}\,\,)$, *the linear operator* $K_{\mathcal{B}}^{\delta }$ *has the cut-off property* $\mathrm {supp}_{\mathcal{T}}\,(K_{\mathcal{B}}^{\delta } v) \subseteq {\mathcal{B}}^{\delta }_{}$ *and the local projection property* $(K_{\mathcal{B}}^{\delta } v)|_{\mathcal{B}} = v|_{\mathcal{B}}$. *Furthermore*, $K_{\mathcal{B}}^{\delta }$ *preserves homogeneous boundary values: For all* $v \in \mathbb {S}^{p,1}_{0}(\mathcal{T}\,\,)$, *there holds* $K_{\mathcal{B}}^{\delta } v \in \mathbb {S}^{p,1}_{0}(\mathcal{T}\,\,)$. *Finally, for every* $v \in \mathbb {S}^{p,1}(\mathcal{T}\,\,)$ *and every* $T \in \mathcal{T}$, *there holds the local stability estimate*

$$\begin{aligned} \Vert K_{\mathcal{B}}^{\delta } v\Vert _{L^{2}(T)} + \delta |K_{\mathcal{B}}^{\delta } v|_{H^{1}(T)}&\lesssim \Vert v\Vert _{L^{2}(T)} + \delta |v|_{H^{1}(T)}. \end{aligned}$$

#### Proof

The cut-off property, the local projection property and the preservation of homogeneous boundary values follow directly from Lemma 3.21 and Lemma 3.18. Finally, let $v \in \mathbb {S}^{p,1}(\mathcal{T}\,\,)$ and $T \in \mathcal{T}$. Note that $\kappa _{\mathcal{B}}^{\delta } v \in \mathbb {S}^{p+1,1}(\mathcal{T}\,\,)$, i.e., we can use the stability estimate from Lemma 3.21:

$$\begin{aligned} \sum _{\ell =0}^{1} \delta ^{\ell } |K_{\mathcal{B}}^{\delta } v|_{H^{\ell }(T)}&\lesssim \sum _{\ell =0}^{1} \delta ^{\ell } |\kappa _{\mathcal{B}}^{\delta } v|_{H^{\ell }(T)} \lesssim \sum _{\ell =0}^{1} \delta ^{\ell } \sum _{i=0}^{\ell } |\kappa _{\mathcal{B}}^{\delta }|_{W^{\ell -i,\infty }(T)} |v|_{H^{i}(T)} \\&{\mathop {\lesssim }\limits ^{\text {Lem.~3.18}}} \sum _{\ell =0}^{1} \delta ^{\ell } \sum _{i=0}^{\ell } \delta ^{i-\ell } |v|_{H^{i}(T)} \lesssim \sum _{\ell =0}^{1} \delta ^{\ell } |v|_{H^{\ell }(T)}. \end{aligned}$$

This finishes the proof. $\square$

### The spaces of locally discrete harmonic functions

In this subsection, we introduce the spaces of *locally discrete harmonic functions*. As we already mentioned in Sect. 3.1, they are chosen for three main reasons: To begin with, they fit in seamlessly with the discrete solution operator $S_{\mathcal{T}}: L^{2}(\varOmega ) \longrightarrow \mathbb {S}^{p,1}_{0}(\mathcal{T}\,\,)$ from Definition 3.8. Furthermore, as specified in Lemma 3.26, they are invariant with respect to the discrete cut-off operators $K_{\mathcal{B}}^{\delta }: \mathbb {S}^{p,1}(\mathcal{T}\,\,) \longrightarrow \mathbb {S}^{p,1}(\mathcal{T}\,\,)$ from Definition 3.23. But most importantly, they contain functions whose $H^1$-norms can be bounded by $L^2$-norms with constants independent of $h_{}$, i.e., a *discrete Caccioppoli inequality* holds.

#### Definition 3.25

For every $\mathcal{B}\subseteq \mathcal{T}$, the space of *locally discrete harmonic functions* is given by

$$\begin{aligned} \mathbb {S}_{\mathrm {harm}}(\mathcal{B}) {:=} \{u \in \mathbb {S}^{p,1}_{0}(\mathcal{T}\,\,)\,|\,\forall v \in \mathbb {S}^{p,1}_{0}(\mathcal{T}\,\,) \,\, \text {with} \,\, \mathrm {supp}_{\mathcal{T}}\,(v) \subseteq \mathcal{B}: a(u,v) = 0\} \subseteq \mathbb {S}^{p,1}_{0}(\mathcal{T}\,\,). \end{aligned}$$

We summarize the first two main features of the spaces $\mathbb {S}_{\mathrm {harm}}(\mathcal{B})$ in the next lemma, namely, their relationships to the discrete solution operator $S_{\mathcal{T}}: L^{2}(\varOmega ) \longrightarrow \mathbb {S}^{p,1}_{0}(\mathcal{T}\,\,)$ and the discrete cut-off operators $K_{\mathcal{B}}^{\delta }: \mathbb {S}^{p,1}(\mathcal{T}\,\,) \longrightarrow \mathbb {S}^{p,1}(\mathcal{T}\,\,)$.

#### Lemma 3.26

*The spaces of locally discrete harmonic functions are nested in the sense*

$$\begin{aligned} \forall \mathcal{B}\subseteq \mathcal{B}^+ \subseteq \mathcal{T}: \quad \quad \mathbb {S}_{\mathrm {harm}}(\mathcal{B}^+) \subseteq \mathbb {S}_{\mathrm {harm}}(\mathcal{B}). \end{aligned}$$

*Furthermore, for all clusters* $\mathcal{B}$, $\mathcal{D}\subseteq \mathcal{T}$ *with* $\mathcal{B}\cap \mathcal{D}= \emptyset$, *the operator* $S_{\mathcal{T}}$ *has the mapping property*

$$\begin{aligned} \forall f \in L^{2}(\varOmega ) \,\, \text {with} \,\, \mathrm {supp}_{\mathcal{T}}\,(f) \subseteq \mathcal{D}: \quad \quad S_{\mathcal{T}}\, f \in \mathbb {S}_{\mathrm {harm}}(\mathcal{B}). \end{aligned}$$

*Finally, for all* $\mathcal{B}\subseteq \mathcal{T}$ *and all* $\delta >0$ *with* $4\sigma _{\mathrm {shp}}^3 h_{\mathcal{B}} \le \delta \lesssim 1$, *we have the invariance*

$$\begin{aligned} \forall u \in \mathbb {S}_{\mathrm {harm}}(\mathcal{B}): \quad \quad K_{\mathcal{B}}^{\delta } u \in \mathbb {S}_{\mathrm {harm}}(\mathcal{B}). \end{aligned}$$

#### Proof

The inclusion $\mathbb {S}_{\mathrm {harm}}(\mathcal{B}^+) \subseteq \mathbb {S}_{\mathrm {harm}}(\mathcal{B})$ follows directly from the definition of the spaces. As for the mapping properties of $S_{\mathcal{T}}$, let $f \in L^{2}(\varOmega )$ with $\mathrm {supp}_{\mathcal{T}}\,(f) \subseteq \mathcal{D}$. Then, for every $v \in \mathbb {S}^{p,1}_{0}(\mathcal{T}\,\,)$ with $\mathrm {supp}_{\mathcal{T}}\,(v) \subseteq \mathcal{B}$, we have

$$\begin{aligned} a(S_{\mathcal{T}}\, f,v) {\mathop {=}\limits ^{\text {Def.~3.8}}} \langle f,v\rangle _{L^{2}(\mathcal{D}\cap \mathcal{B})} {\mathop {=}\limits ^{\mathcal{B}\cap \mathcal{D}= \emptyset }} 0. \end{aligned}$$

Finally, consider a function $u \in \mathbb {S}_{\mathrm {harm}}(\mathcal{B})$ and an arbitrary $v \in \mathbb {S}^{p,1}_{0}(\mathcal{T}\,\,)$ with $\mathrm {supp}_{\mathcal{T}}\,(v) \subseteq \mathcal{B}$. Then,

$$\begin{aligned} a(K_{\mathcal{B}}^{\delta } u,v)&{\mathop {=}\limits ^{\text {Def.~2.1}}}&\langle a_1 \nabla K_{\mathcal{B}}^{\delta } u,\nabla _{} v\rangle _{L^{2}(\mathcal{B})} + \langle a_2 \cdot \nabla K_{\mathcal{B}}^{\delta } u,v\rangle _{L^{2}(\mathcal{B})} + \langle a_3 K_{\mathcal{B}}^{\delta } u,v\rangle _{L^{2}(\mathcal{B})} \\&{\mathop {=}\limits ^{\text {Lem.~3.24}}}&\langle a_1 \nabla _{} u,\nabla _{} v\rangle _{L^{2}(\mathcal{B})} + \langle a_2 \cdot \nabla _{} u,v\rangle _{L^{2}(\mathcal{B})} + \langle a_3 u,v\rangle _{L^{2}(\mathcal{B})} \\= & \,{} a(u,v) \\= &\, {} 0. \end{aligned}$$

This gives $K_{\mathcal{B}}^{\delta } u \in \mathbb {S}_{\mathrm {harm}}(\mathcal{B})$, which concludes the proof. $\square$

Next, we turn our attention to the *discrete Caccioppoli inequality*. In a nutshell, it will allow us to bound an $H^1$-norm on a cluster $\mathcal{B}\subseteq \mathcal{T}$ by an $L^2$-norm on the slightly larger cluster ${\mathcal{B}}^{\delta }_{}$. Obviously, this can only be true for a certain subspace $V \subseteq \mathbb {S}^{p,1}(\mathcal{T}\,\,)$. In our setting, this is the space of locally discrete harmonic functions $\mathbb {S}_{\mathrm {harm}}({\mathcal{B}}^{\delta }_{})$ from Definition 3.25. We can interpret the discrete Caccioppoli inequality as an improved version of the inverse inequality from Lemma 3.20, which bounds an $H^1$-seminorm by an $L^2$-norm, too. This time, however, the prefactor $h_{}$ of the $H^1$-seminorm can be increased to a (possibly much) bigger parameter $\delta \gg h_{}$.

#### Lemma 3.27

*Let* $\mathcal{B}\subseteq \mathcal{T}$ *and* $\delta >0$ *with* $4\sigma _{\mathrm {shp}}^3 h_{\mathcal{B}} \le \delta \lesssim 1$. *Then, for every* $u \in \mathbb {S}_{\mathrm {harm}}({\mathcal{B}}^{\delta }_{})$, *there holds the* *discrete Caccioppoli inequality*

$$\begin{aligned} \delta |u|_{H^{1}(\mathcal{B})} \lesssim \Vert u\Vert _{L^{2}({\mathcal{B}}^{\delta }_{})}. \end{aligned}$$

#### Proof

First off, by induction on $p \ge 1$ we show the following estimate: For every $\kappa _{}^{} \in \mathbb {S}^{1,1}(\mathcal{T}\,\,)$, $u \in \mathbb {S}^{p,0}(\mathcal{T}\,\,)$ and $T \in \mathcal{T}$,

3.1 $$\begin{aligned} h_{T}^{p+1} |{\kappa _{}^{}}^2 u|_{H^{p+1}(T)} \lesssim h_{T}^2 |\kappa _{}^{}|_{W^{1,\infty }(T)} ( \Vert u \nabla _{} \kappa _{}^{}\Vert _{L^{2}(T)} + \Vert \kappa _{}^{} \nabla _{} u\Vert _{L^{2}(T)} ). \end{aligned}$$

In the base case $p=1$, the second order derivatives in $|{\kappa _{}^{}}^2 u|_{H^{2}(T)}$ can be computed explicitly. Since $\kappa _{}^{}$, $u \in \mathbb {P}^{1}(T)$, the terms containing $\mathrm {D}^{\alpha } \kappa _{}^{}$ or $\mathrm {D}^{\alpha } u$ with $|\alpha |=2$ are not present. In the induction step $p \mapsto p+1$, we estimate $|{\kappa _{}^{}}^2 u|_{H^{p+2}(T)} \lesssim \sum _i |\kappa _{}^{} (\partial _{i}^{} \kappa _{}^{}) u|_{H^{p+1}(T)} + |{\kappa _{}^{}}^2 (\partial _{i}^{} u)|_{H^{p+1}(T)}$. For the first summand, we use the inverse inequality Lemma 3.20 and get $|\kappa _{}^{} (\partial _{i}^{} \kappa _{}^{}) u|_{H^{p+1}(T)} \lesssim h_{T}^{-p} |\kappa _{}^{} (\partial _{i}^{} \kappa _{}^{}) u|_{H^{1}(T)}$. Again, we can expand the derivatives explicitly and cancel all terms containing second order derivatives of $\kappa _{}^{} \in \mathbb {P}^{1}(T)$. The second summand is directly amenable to the induction hypothesis, i.e., (3.1): $|{\kappa _{}^{}}^2 (\partial _{i}^{} u)|_{H^{p+1}(T)} \lesssim h_{T}^{1-p} |\kappa _{}^{}|_{W^{1,\infty }(T)} ( \Vert (\partial _{i}^{} u) \nabla _{} \kappa _{}^{}\Vert _{L^{2}(T)} + \Vert \kappa _{}^{} \nabla _{}(\partial _{i}^{} u)\Vert _{L^{2}(T)} )$. Since $\nabla _{} \kappa _{}^{} \equiv \mathrm {const}$, we have

$$\Vert (\partial _{i}^{} u) \nabla _{} \kappa _{}^{}\Vert _{L^{2}(T)} = |\nabla _{} \kappa _{}^{}| \Vert \partial _{i}^{} u\Vert _{L^{2}(T)} \lesssim h_{T}^{-1} |\nabla _{} \kappa _{}^{}| \Vert u\Vert _{L^{2}(T)} = h_{T}^{-1}\Vert u \nabla _{} \kappa _{}^{}\Vert _{L^{2}(T)}.$$

The term $\Vert \kappa _{}^{} \nabla _{}(\partial _{i}^{} u)\Vert _{L^{2}(T)}$ can be treated with the identity $\kappa _{}^{} \nabla _{}(\partial _{i}^{} u) = \partial _i(\kappa _{}^{} \nabla _{} u) - (\partial _{i}^{} \kappa _{}^{}) \nabla _{} u$ and the inverse inequality Lemma 3.20 using the same arguments. Multiplication with $h_{T}^{p+2}$ then proves the induction step.

Let us turn our attention to the discrete Caccioppoli inequality itself. To this end, let $\mathcal{B}\subseteq \mathcal{T}$ and $\delta >0$ with $4\sigma _{\mathrm {shp}}^3 h_{\mathcal{B}} \le \delta \lesssim 1$. We denote by $\kappa _{}^{} {:=} \kappa _{\mathcal{B}}^{\delta } \in \mathbb {S}^{1,1}(\mathcal{T}\,\,)$ the discrete cut-off function from Lemma 3.18 and by $I_{\mathcal{T}}^p: C^{0}_{\mathrm {pw}}(\mathcal{T}\,\,) \longrightarrow \mathbb {S}^{p,0}(\mathcal{T}\,\,)$ the Lagrange interpolation operator from Definition 3.19. Furthermore, let $u \in \mathbb {S}_{\mathrm {harm}}({\mathcal{B}}^{\delta }_{})$. The key step of the proof is to exploit the orthogonality $a(u,v) = 0$ for some carefully chosen test function $v \in \mathbb {S}^{p,1}_{0}(\mathcal{T}\,\,)$ with $\mathrm {supp}_{\mathcal{T}}\,(v) \subseteq {\mathcal{B}}^{\delta }_{}$. From Lemmas 3.21 and 3.18 we know that $v {:=} I_{\mathcal{T}}^p({\kappa _{}^{}}^2 u)$ satisfies both $v \in \mathbb {S}^{p,1}_{0}(\mathcal{T}\,\,)$ and $\mathrm {supp}_{\mathcal{T}}\,(v) \subseteq \mathrm {supp}_{\mathcal{T}}\,(\kappa _{}^{}) \subseteq {\mathcal{B}}^{\delta }_{}$, i.e., we can use *v* as a test function. This results in the following bound:

$$\begin{aligned} a(u,{\kappa _{}^{}}^2 u)&= a(u,{\kappa _{}^{}}^2 u-v) = a(u,(\mathrm {id}- I_{\mathcal{T}}^p)({\kappa _{}^{}}^2 u)) \\&{\mathop {\lesssim }\limits ^{\text {Def.~2.1}}} \sum _{T \in {\mathcal{B}}^{\delta }_{}} \Vert u\Vert _{H^{1}(T)} \Vert (\mathrm {id}- I_{\mathcal{T}}^p)({\kappa _{}^{}}^2 u)\Vert _{H^{1}(T)} \\&{\mathop {\lesssim }\limits ^{\text {Lem.~3.21}}} \sum _{T \in {\mathcal{B}}^{\delta }_{}} \Vert u\Vert _{H^{1}(T)} h_{T}^p |{\kappa _{}^{}}^2 u|_{H^{p+1}(T)} \\&\lesssim |\kappa _{}^{}|_{W^{1,\infty }(\varOmega )} \sum _{T \in {\mathcal{B}}^{\delta }_{}} h_{T} \Vert u\Vert _{H^{1}(T)} ( \Vert u \nabla _{} \kappa _{}^{}\Vert _{L^{2}(T)} + \Vert \kappa _{}^{} \nabla _{} u\Vert _{L^{2}(T)} ) \\&{\mathop {\lesssim }\limits ^{\text {Lem.~3.20}}} |\kappa _{}^{}|_{W^{1,\infty }(\varOmega )} \sum _{T \in {\mathcal{B}}^{\delta }_{}} \Vert u\Vert _{L^{2}(T)} (\Vert u \nabla _{} \kappa _{}^{}\Vert _{L^{2}(T)} + \Vert \kappa _{}^{} \nabla _{} u\Vert _{L^{2}(T)}) \\&{\mathop {\lesssim }\limits ^{\text {C.Sch.}}} |\kappa _{}^{}|_{W^{1,\infty }(\varOmega )} \Vert u\Vert _{L^{2}({\mathcal{B}}^{\delta }_{})} ( \Vert u \nabla _{} \kappa _{}^{}\Vert _{L^{2}(\varOmega )} + \Vert \kappa _{}^{} \nabla _{} u\Vert _{L^{2}(\varOmega )} ). \end{aligned}$$

On the other hand, using the coercivity of the PDE coefficient $a_1$ in the bilinear form $a(\cdot ,\cdot )$, cf. Sect. 2.1, we can expand the term $a(u,{\kappa _{}^{}}^2 u)$ and rearrange the summands:

$$\begin{aligned}&\Vert \kappa _{}^{} \nabla _{} u\Vert _{L^{2}(\varOmega )}^2 \lesssim \langle a_1 \kappa _{}^{} \nabla _{} u,\kappa _{}^{} \nabla _{} u\rangle _{L^{2}(\varOmega )} \\&\quad {\mathop {=}\limits ^{\text {Def.~2.1}}} a(u,{\kappa _{}^{}}^2 u) - 2\langle a_1 \kappa _{}^{} \nabla _{} u,u \nabla _{} \kappa _{}^{}\rangle _{L^{2}(\varOmega )} - \langle a_2 \cdot \nabla _{} u,{\kappa _{}^{}}^2 u\rangle _{L^{2}(\varOmega )} - \langle a_3 u,{\kappa _{}^{}}^2 u\rangle _{L^{2}(\varOmega )} \\&\quad \lesssim |\kappa _{}^{}|_{W^{1,\infty }(\varOmega )} \Vert u\Vert _{L^{2}({\mathcal{B}}^{\delta }_{})} ( \Vert u \nabla _{} \kappa _{}^{}\Vert _{L^{2}(\varOmega )} + \Vert \kappa _{}^{} \nabla _{} u\Vert _{L^{2}(\varOmega )} ) \\&\qquad \qquad + \Vert \kappa _{}^{} \nabla _{} u\Vert _{L^{2}(\varOmega )} \Vert u \nabla _{} \kappa _{}^{}\Vert _{L^{2}(\varOmega )} + \Vert \kappa _{}^{} \nabla _{} u\Vert _{L^{2}(\varOmega )} \Vert \kappa _{}^{} u\Vert _{L^{2}(\varOmega )} + \Vert \kappa _{}^{} u\Vert _{L^{2}(\varOmega )}^2 \\&\quad {\mathop {\le }\limits ^{\forall \varepsilon >0}} C_\varepsilon \Vert \kappa _{}^{}\Vert _{W^{1,\infty }(\varOmega )}^2 \Vert u\Vert _{L^{2}({\mathcal{B}}^{\delta }_{})}^2 + \varepsilon \Vert \kappa _{}^{} \nabla _{} u\Vert _{L^{2}(\varOmega )}^2. \end{aligned}$$

Finally, since the parameter $\varepsilon >0$ from Young’s inequality can be chosen arbitrarily small, we can absorb the last summand of the right-hand side in the left-hand side of the overall inequality. We end up with

$$\begin{aligned} |u|_{H^{1}(\mathcal{B})} {\mathop {\le }\limits ^{\kappa _{}^{}|_{\mathcal{B}} \equiv 1}} \Vert \kappa _{}^{} \nabla _{} u\Vert _{L^{2}(\varOmega )} \lesssim \Vert \kappa _{}^{}\Vert _{W^{1,\infty }(\varOmega )} \Vert u\Vert _{L^{2}({\mathcal{B}}^{\delta }_{})} {\mathop {\lesssim }\limits ^{\text {Lem.~3.18}}} \frac{1}{\delta } \Vert u\Vert _{L^{2}({\mathcal{B}}^{\delta }_{})}. \end{aligned}$$

This concludes the proof of the discrete Caccioppoli inequality. $\square$

### The single- and multi-step coarsening operators

In this subsection, we do the actual work in the construction of the subspace $V_{\mathcal{B},\mathcal{D},L} \subseteq \mathbb {S}^{p,1}_{0}(\mathcal{T}\,\,)$ from Sect. 3.1. We design the so called *single-* and *multi-step coarsening operators*. Given $\mathcal{B}\subseteq \mathcal{T}$, $\delta >0$ and $u \in \mathbb {S}_{\mathrm {harm}}({\mathcal{B}}^{\delta }_{})$, the single-step coarsening operator $Q_{\mathcal{B}}^{\delta }$ produces a “coarse” approximation $Q_{\mathcal{B}}^{\delta } u \in \mathbb {S}_{\mathrm {harm}}(\mathcal{B})$ with an error $\Vert u - Q_{\mathcal{B}}^{\delta } u\Vert _{L^{2}(\mathcal{B})} \le 2^{-1} \Vert u\Vert _{L^{2}({\mathcal{B}}^{\delta }_{})}$. The prefactor $2^{-1} \in (0,1)$ is essential, as it produces an exponential factor $2^{-L}$ when $L \in \mathbb {N}$ single-step coarsening operators are combined in a specific manner. This is precisely the idea behind the multi-step coarsening operator $Q_{\mathcal{B}}^{\delta ,L}$. Given a function $u \in \mathbb {S}_{\mathrm {harm}}({\mathcal{B}}^{\delta L}_{})$, it produces a “coarse” approximation $Q_{\mathcal{B}}^{\delta ,L} u \in \mathbb {S}_{\mathrm {harm}}(\mathcal{B})$ with an error $\Vert u - Q_{\mathcal{B}}^{\delta ,L} u\Vert _{L^{2}(\mathcal{B})} \le 2^{-L} \Vert u\Vert _{L^{2}({\mathcal{B}}^{\delta L}_{})}$.

As our construction of the single-step coarsening operator in Theorem 3.31 is quite technical, we would like to reveal the underlying ideas first: Assume for a moment that $\mathcal{T}$ is uniform, i.e., $h_{\mathcal{T}} \eqsim h_{\min ,\mathcal{T}}$. Then, a function $u \in \mathbb {S}_{\mathrm {harm}}({\mathcal{B}}^{\delta }_{})$ is described by up to $\mathrm {dim}\,\mathbb {S}^{p,0}(\mathcal{T}\,\,) \eqsim \# \mathcal{T} \eqsim h_{\mathcal{T}}^{-d}$ degrees of freedom. In order to reduce this number, we could approximate $u \approx \Pi _{\mathcal{S}}^p u \in \mathbb {S}^{p,0}(\mathcal{S})$, where $\mathcal{S}\subseteq \mathrm {Pow}(\varOmega )$ is a second uniform mesh and where $\Pi _{\mathcal{S}}^p: L^{2}(\varOmega ) \longrightarrow \mathbb {S}^{p,0}(\mathcal{S})$ is some kind of approximation operator. As long as $\mathcal{S}$ is coarser than $\mathcal{T}$, i.e. $h_{\mathcal{S}} \gtrsim h_{\mathcal{T}}$, this provides a reduction of the dimension. On the other hand, the typical error bound $\Vert u - \Pi _{\mathcal{S}}^p u\Vert _{L^{2}(\varOmega )} \lesssim H |u|_{H^{1}(\varOmega )}$ involves an $H^1$-norm on the right-hand side. In order to get rid of the $H^1$-norm, we want to apply the discrete Caccioppoli inequality, Lemma 3.27. For this to work, however, we first need to reduce the global quantity $H |u|_{H^{1}(\varOmega )}$ to the local quantity $H |u|_{H^{1}(\mathcal{B})}$. This can be done using the discrete cut-off operator $K_{\mathcal{B}}^{\delta }$ from Definition 3.23. Finally, the combined operator $\Pi _{\mathcal{S}}^p K_{\mathcal{B}}^{\delta }: \mathbb {S}_{\mathrm {harm}}({\mathcal{B}}^{\delta }_{}) \longrightarrow \mathbb {S}^{p,0}(\mathcal{S})$ only lacks one more thing: It does not necessarily map into the space $\mathbb {S}_{\mathrm {harm}}(\mathcal{B})$, which is a critical requirement, because we want to iterate the argument by plugging the remainder $\tilde{u} {:=} \,u - Q_{\mathcal{B}}^{\delta } u$ of one single-step coarsening operator into another one. Thankfully, we can simply append the orthogonal projection $P_{\mathcal{B}}: L^{2}(\varOmega ) \longrightarrow \mathbb {S}_{\mathrm {harm}}(\mathcal{B})$ without losing any of the aforementioned properties.

In the next lemma we provide a construction for the second, coarser mesh $\mathcal{S}\subseteq \mathrm {Pow}(\varOmega )$:

#### Lemma 3.28

*Let* $\mathcal{S}_0 \subseteq \mathrm {Pow}(\varOmega )$ *be an arbitrary mesh and* $(\mathcal{S}_\ell )_{\ell \in \mathbb {N}_0}$ *be the corresponding sequence of uniform refinements*. *For every* $H>0$, *there exists an* $\mathcal{S}\in (\mathcal{S}_\ell )_{\ell \in \mathbb {N}_0}$ *with* $\sigma _{\mathrm {shp}}(\mathcal{S}) = C(\mathcal{S}_0)$ *and* $C(\mathcal{S}_0) H \le h_{\min ,\mathcal{S}} \le h_{\mathcal{S}} \le H$. *In particular*, $\mathcal{S}$ *is uniform in the sense of Definition* 3.2.

#### Proof

There hold the relations $h_{\mathcal{S}_\ell } = 2^{-\ell } h_{\mathcal{S}_0}$ and $h_{\min ,\mathcal{S}_\ell } = 2^{-\ell } h_{\min ,\mathcal{S}_0}$. For any given $H>0$, we choose the mesh $\mathcal{S}{:=} \mathcal{S}_L$, where $L \in \mathbb {N}_0$ is the minimal level satisfying $h_{\mathcal{S}_L} \le H$. In particular, there also holds the lower bound $H < h_{\mathcal{S}_{L-1}} = 2^{-(L-1)} h_{\mathcal{S}_0} = 2 h_{\mathcal{S}_0} h_{\min ,\mathcal{S}_0}^{-1} h_{\min ,\mathcal{S}_L} = C(\mathcal{S}_0) h_{\min ,\mathcal{S}_L}$. $\square$

The additional mesh $\mathcal{S}\subseteq \mathrm {Pow}(\varOmega )$ does not need to be aligned with the original mesh $\mathcal{T}\subseteq \mathrm {Pow}(\varOmega )$ at all. The output of the cut-off operator $K_{\mathcal{B}}^{\varepsilon }$ is just an element of $\mathbb {S}^{p,1}_{0}(\mathcal{T}\,\,) \subseteq H^{1}(\varOmega )$, so we need an operator $\Pi _{\mathcal{S}}: H^{1}(\varOmega ) \longrightarrow \mathbb {S}^{q,0}(\mathcal{S})$ for some $q \ge 0$. Also, in the case $\mathcal{S}= \mathcal{T}$ the operator should act like a projection on functions from $\mathbb {S}^{p,1}_{0}(\mathcal{T}\,\,)$. The simplest solution for these demands is the $L^{2}(\varOmega )$-*orthogonal projection*.

#### Definition 3.29

We denote by $\Pi _{\mathcal{S}}^p: L^{2}(\varOmega ) \longrightarrow \mathbb {S}^{p,0}(\mathcal{S})$ the (global) orthogonal projection from $L^{2}(\varOmega )$ onto the closed subspace $\mathbb {S}^{p,0}(\mathcal{S})$.

In fact, $\Pi _{\mathcal{S}}^p$ coincides with the piecewise $L^2$-orthogonal projection on the mesh $\mathcal{S}$. This results in desirable *local* properties and bounds.

#### Lemma 3.30

*The linear operator* $\Pi _{\mathcal{S}}^p$ *has a local projection property: For every cluster* $\mathcal{B}\subseteq \mathcal{S}$ *and every function* $v \in L^{2}(\varOmega )$ *with* $v \in \mathbb {S}^{p,0}(\mathcal{B})$, *there holds* $(\Pi _{\mathcal{S}}^p v)|_{\mathcal{B}} = v|_{\mathcal{B}}$. *Furthermore*, $\Pi _{\mathcal{S}}^p$ *preserves supports: For every* $v \in L^{2}(\varOmega )$, *we have* $\mathrm {supp}_{\mathcal{S}}(\Pi _{\mathcal{S}}^p v) \subseteq \mathrm {supp}_{\mathcal{S}}(v)$. *Finally, for every* $k \in \{0,\dots ,p+1\}$, *there hold the stability and error estimates*

$$\begin{aligned} \begin{array}{lrcl} \forall v \in H^{k}_{\mathrm {pw}}(\mathcal{S}): \forall S \in \mathcal{S}: \quad &{} \sum\limits_{\ell =0}^{k} h_{S}^{\ell } |\mathop{\Pi}_{\mathcal{S}}^p v|_{H^{\ell }(S)} &{}\lesssim &{} \sum\limits_{\ell =0}^{k} h_{S}^{\ell } |v|_{H^{\ell }(S)}, \\ &{} \sum\limits_{\ell =0}^{k} h_{S}^{\ell } |(\mathrm {id}- \Pi _{\mathcal{S}}^p)(v)|_{H^{\ell }(S)} &{}\lesssim &{} h_{S}^k |v|_{H^{k}(S)}. \end{array} \end{aligned}$$

Now, we have all the ingredients for the construction of the *single-step coarsening operator*.

#### Theorem 3.31

*Let* $\mathcal{T}\subseteq \mathrm {Pow}(\varOmega )$ *be a mesh of locally bounded cardinality*. *Furthermore, let* $\mathcal{B}\subseteq \mathcal{T}$ *and* $\delta >0$ *with* $\delta \lesssim 1$. *Then, there exists a linear* *single-step coarsening operator*

$$\begin{aligned} Q_{\mathcal{B}}^{\delta }: \mathbb {S}_{\mathrm {harm}}({\mathcal{B}}^{\delta }_{}) \longrightarrow \mathbb {S}_{\mathrm {harm}}(\mathcal{B}) \end{aligned}$$

*of rank*

$$\begin{aligned} \mathrm {rank}(Q_{\mathcal{B}}^{\delta }) \lesssim \bigg (1 + \frac{\mathrm {diam}_{\mathcal{T}}(\mathcal{B})}{\delta } \bigg )^{d \sigma _{\mathrm {card}}} \end{aligned}$$

*that satisfies the following approximation property*: *For every* $u \in \mathbb {S}_{\mathrm {harm}}({\mathcal{B}}^{\delta }_{})$,

$$\begin{aligned} \Vert u - Q_{\mathcal{B}}^{\delta } u\Vert _{L^{2}(\mathcal{B})} \le \frac{1}{2} \Vert u\Vert _{L^{2}({\mathcal{B}}^{\delta }_{})}. \end{aligned}$$

#### Proof

Let $\mathcal{B}\subseteq \mathcal{T}$ and $\delta >0$ with $\delta \lesssim 1$. For the construction of $Q_{\mathcal{B}}^{\delta }$ we need three operators: First, we use the discrete cut-off operator $K_{\mathcal{B}}^{\varepsilon }: \mathbb {S}^{p,1}(\mathcal{T}\,\,) \longrightarrow \mathbb {S}^{p,1}(\mathcal{T}\,\,)$ from Definition 3.23 with some carefully chosen parameter $\varepsilon >0$. Second, we apply the piecewise orthogonal projection $\Pi _{\mathcal{S}}^p: L^{2}(\varOmega ) \longrightarrow \mathbb {S}^{p,0}(\mathcal{S})$ from Definition 3.29 on some suitable mesh $\mathcal{S}\subseteq \mathrm {Pow}(\varOmega )$. Third, the result is mapped back into the space $\mathbb {S}_{\mathrm {harm}}(\mathcal{B})$ via the orthogonal projection $P_{\mathcal{B}}: L^{2}(\varOmega ) \longrightarrow \mathbb {S}_{\mathrm {harm}}(\mathcal{B})$.

For the precise choice of $\varepsilon$ and $\mathcal{S}$ we have to distinguish between two cases: In the more involved case $\delta \ge 20\sigma _{\mathrm {shp}}^7 h_{\mathcal{B}}$ we choose $\varepsilon {:=} \delta /(5\sigma _{\mathrm {shp}}^4) \ge 4\sigma _{\mathrm {shp}}^3 h_{\mathcal{B}}$ and use the uniform mesh $\mathcal{S}\subseteq \mathrm {Pow}(\varOmega )$ from Lemma 3.28 with $h_{\mathcal{S}} \eqsim h_{\min ,\mathcal{S}} \eqsim H$, where the parameter $H>0$ will be specified during the proof. In the degenerate case $\delta < 20\sigma _{\mathrm {shp}}^7 h_{\mathcal{B}}$ we set $\varepsilon {:=} 4\sigma _{\mathrm {shp}}^3 h_{\mathcal{B}}$ and use the mesh $\mathcal{S}{:=} \mathcal{T}$ itself.

We define the asserted operator as

$$\begin{aligned} Q_{\mathcal{B}}^{\delta } {:=} P_{\mathcal{B}} \Pi _{\mathcal{S}}^p K_{\mathcal{B}}^{\varepsilon }: \mathbb {S}_{\mathrm {harm}}({\mathcal{B}}^{\delta }_{}) \longrightarrow \mathbb {S}_{\mathrm {harm}}(\mathcal{B}). \end{aligned}$$

*The case* $\delta \ge 20\sigma _{\mathrm {shp}}^7 h_{\mathcal{B}}$: Let $u \in \mathbb {S}_{\mathrm {harm}}({\mathcal{B}}^{\delta }_{})$. By Lemma 3.15 we have $h_{{\mathcal{B}}^{\varepsilon }_{}} \le \max \{h_{\mathcal{B}}, \sigma _{\mathrm {shp}}\varepsilon \}$, and the assumption on $\delta$ implies $h_{\mathcal{B}}\le \delta /(20\sigma _{\mathrm {shp}}^7) = \varepsilon /(4\sigma _{\mathrm {shp}}^3)<\varepsilon$, so the maximum in the previous estimate is attained at $\sigma _{\mathrm {shp}}\varepsilon$. Therefore, the parameter $\alpha {:=} 4\sigma _{\mathrm {shp}}^4 \varepsilon$ satisfies $4\sigma _{\mathrm {shp}}^3 h_{{\mathcal{B}}^{\varepsilon }_{}} \le \alpha \lesssim 1$. In particular, we can apply the discrete Caccioppoli inequality to the set ${\mathcal{B}}^{\varepsilon }_{}$ and the parameter $\alpha$. Since $\delta \eqsim \alpha$, this gives the stability estimate for the cut-off operator $K_{\mathcal{B}}^{\varepsilon }$

$$\begin{aligned} \sum _{\ell =0}^{1} \delta ^\ell |K_{\mathcal{B}}^{\varepsilon } u|_{H^{\ell }(\varOmega )} {\mathop {\lesssim }\limits ^{\text {Lem.~3.24}}} \sum _{\ell =0}^{1} \alpha ^\ell |u|_{H^{\ell }({\mathcal{B}}^{\varepsilon }_{})} {\mathop {\lesssim }\limits ^{\text {Lem.~3.27}}} \Vert u\Vert _{L^{2}({\mathcal{B}}^{\varepsilon +\alpha }_{})} {\mathop {\le }\limits ^{\varepsilon +\alpha \le \delta }} \Vert u\Vert _{L^{2}({\mathcal{B}}^{\delta }_{})}. \end{aligned}$$

From Lemmas 3.26 and 3.24 we know that $K_{\mathcal{B}}^{\varepsilon } u \in \mathbb {S}_{\mathrm {harm}}(\mathcal{B})$, hence $P_{\mathcal{B}} K_{\mathcal{B}}^{\varepsilon } u = K_{\mathcal{B}}^{\varepsilon } u$. We conclude $u|_{\mathcal{B}} = (K_{\mathcal{B}}^{\varepsilon } u)|_{\mathcal{B}} = (P_{\mathcal{B}} K_{\mathcal{B}}^{\varepsilon } u)|_{\mathcal{B}}$ and thus

$$\begin{aligned} \begin{array}{rclcl} \Vert u - Q_{\mathcal{B}}^{\delta } u\Vert _{L^{2}(\mathcal{B})} &{}=&{} \Vert P_{\mathcal{B}} K_{\mathcal{B}}^{\varepsilon } u - P_{\mathcal{B}} \Pi _{\mathcal{S}}^p K_{\mathcal{B}}^{\varepsilon } u\Vert _{L^{2}(\mathcal{B})} &{}\le &{} \Vert P_{\mathcal{B}} (\mathrm {id}- \Pi _{\mathcal{S}}^p)(K_{\mathcal{B}}^{\varepsilon } u)\Vert _{L^{2}(\varOmega )} \\ &{}\le &{} \Vert (\mathrm {id}- \Pi _{\mathcal{S}}^p)(K_{\mathcal{B}}^{\varepsilon } u)\Vert _{L^{2}(\varOmega )} &{}{\mathop {\lesssim }\limits ^{\text {Lem.~3.30}}}&{} H |K_{\mathcal{B}}^{\varepsilon } u|_{H^{1}(\varOmega )} \\ &{}\lesssim &{} \frac{H}{\delta } \Vert u\Vert _{L^{2}({\mathcal{B}}^{\delta }_{})}. \end{array} \end{aligned}$$

In particular, we can choose $H \eqsim \delta > 0$ small enough to establish the asserted error bound.

*The case* $\delta < 20\sigma _{\mathrm {shp}}^7 h_{\mathcal{B}}$: Again let $u \in \mathbb {S}_{\mathrm {harm}}({\mathcal{B}}^{\delta }_{})$. Exploiting $\mathcal{S}= \mathcal{T}$ and Lemma 3.24, the operator $Q_{\mathcal{B}}^{\delta }$ reduces to $Q_{\mathcal{B}}^{\delta } u = P_{\mathcal{B}} \Pi _{\mathcal{T}}^p K_{\mathcal{B}}^{\varepsilon } u = P_{\mathcal{B}} K_{\mathcal{B}}^{\varepsilon } u = K_{\mathcal{B}}^{\varepsilon } u$. Consequently, the error bound becomes trivial:

$$\begin{aligned} \Vert u - Q_{\mathcal{B}}^{\delta } u\Vert _{L^{2}(\mathcal{B})} = \Vert u - K_{\mathcal{B}}^{\varepsilon } u\Vert _{L^{2}(\mathcal{B})} = \Vert u - u\Vert _{L^{2}(\mathcal{B})} = 0. \end{aligned}$$

To find a good upper bound for the rank of $Q_{\mathcal{B}}^{\delta }$, the locally bounded cardinality of $\mathcal{S}$ is crucial. In the case $\delta \ge 20\sigma _{\mathrm {shp}}^7 h_{\mathcal{B}}$ the mesh $\mathcal{S}$ is uniform and thus of locally bounded cardinality (cf. Lemma 3.3). In the case $\delta < 20\sigma _{\mathrm {shp}}^7 h_{\mathcal{B}}$ we chose $\mathcal{S}= \mathcal{T}$, which has locally bounded cardinality by assumption.

Next, we abbreviate $B {:=} \bigcup {\mathcal{B}}^{\varepsilon }_{} \subseteq \mathbb {R}^d$ and compute a common lower bound for $h_{\mathcal{S}(B)}$: In the case $\delta \ge 20\sigma _{\mathrm {shp}}^7 h_{\mathcal{B}}$ we have $h_{\mathcal{B}} \lesssim \delta$ and $\varepsilon \lesssim \delta$ and $H \eqsim \delta$ by our choice of the parameters. With Lemma 3.28, this implies $h_{\mathcal{B}} + \varepsilon + \delta \lesssim \delta \eqsim H \eqsim h_{\min ,\mathcal{S}} \le h_{\mathcal{S}(B)}$. In the case $\delta < 20\sigma _{\mathrm {shp}}^7 h_{\mathcal{B}}$, using $\varepsilon \lesssim \delta$ we get in a similar way $h_{\mathcal{B}} + \varepsilon + \delta \lesssim h_{\mathcal{B}} \le h_{\mathcal{T}({\mathcal{B}}^{\varepsilon }_{})} = h_{\mathcal{S}(B)}$ as well.

For every $u \in \mathbb {S}_{\mathrm {harm}}({\mathcal{B}}^{\delta }_{})$ we know from Lemmas 3.30 and 3.24 that $\mathrm {supp}_{\mathcal{S}}(\Pi _{\mathcal{S}}^p K_{\mathcal{B}}^{\varepsilon } u) \subseteq \mathrm {supp}_{\mathcal{S}}(K_{\mathcal{B}}^{\varepsilon } u) \subseteq \mathcal{S}(B)$. This results in the estimate

$$\begin{aligned}&\mathrm {rank}(Q_{\mathcal{B}}^{\delta }) \le \mathrm {dim}\,\{v \in \mathbb {S}^{p,0}(\mathcal{S})\,|\,\mathrm {supp}_{\mathcal{S}}(v) \subseteq \mathcal{S}(B)\} \eqsim \# \mathcal{S}(B) \\&\quad {\mathop {\lesssim }\limits ^{\text {Def.~2.4}}} ( 1 + h_{\mathcal{S}(B)}^{-1} \mathrm {diam}_{\mathcal{S}}(\mathcal{S}(B)) )^{d\sigma _{\mathrm {card}}} {\mathop {\lesssim }\limits ^{\text {Lem.~3.15}}} ( 1 + h_{\mathcal{S}(B)}^{-1}(\mathrm {diam}_{\mathcal{T}}(\mathcal{B}) + h_{\mathcal{B}} + \varepsilon ) )^{d\sigma _{\mathrm {card}}} \\&\quad {\mathop {\lesssim }\limits ^{h_{\mathcal{B}} + \varepsilon \lesssim h_{\mathcal{S}(B)}}} ( 1 + h_{\mathcal{S}(B)}^{-1} \mathrm {diam}_{\mathcal{T}}(\mathcal{B}) )^{d\sigma _{\mathrm {card}}} {\mathop {\lesssim }\limits ^{\delta \lesssim h_{\mathcal{S}(B)}}} ( 1 + \delta ^{-1} \mathrm {diam}_{\mathcal{T}}(\mathcal{B}) )^{d\sigma _{\mathrm {card}}}, \end{aligned}$$

which finishes the proof. $\square$

With the single-step coarsening operator at hand, we can iterate to obtain exponential convergence.

#### Theorem 3.32

*Let* $\mathcal{T}\subseteq \mathrm {Pow}(\varOmega )$ *be a mesh of locally bounded cardinality*. *Furthermore, let* $\mathcal{B}\subseteq \mathcal{T}$ *and* $\delta >0$ *with* $\delta \lesssim 1$. *Then, for every* $L \in \mathbb {N}$, *there exists a linear* *multi-step coarsening operator*

$$\begin{aligned} Q_{\mathcal{B}}^{\delta ,L}: \mathbb {S}_{\mathrm {harm}}({\mathcal{B}}^{\delta L}_{}) \longrightarrow \mathbb {S}_{\mathrm {harm}}(\mathcal{B}) \end{aligned}$$

*of rank*

$$\begin{aligned} \mathrm {rank}(Q_{\mathcal{B}}^{\delta ,L}) \lesssim \bigg (L + \frac{\mathrm {diam}_{\mathcal{T}}(\mathcal{B})}{\delta } \bigg )^{d\sigma _{\mathrm {card}}+1} \end{aligned}$$

*that satisfies the following approximation property: For every* $u \in \mathbb {S}_{\mathrm {harm}}({\mathcal{B}}^{\delta L}_{})$, *there holds*

$$\begin{aligned} \Vert u - Q_{\mathcal{B}}^{\delta ,L} u\Vert _{L^{2}(\mathcal{B})} \le 2^{-L} \Vert u\Vert _{L^{2}({\mathcal{B}}^{\delta L}_{})}. \end{aligned}$$

#### Proof

Let $\mathcal{B}\subseteq \mathcal{T}$ and $\delta >0$ with $\delta \lesssim 1$ as well as $L \in \mathbb {N}$. We define a sequence of nested element sets $\mathcal{B}\subseteq \mathcal{B}_0 \subseteq \dots \subseteq \mathcal{B}_L \subseteq {\mathcal{B}}^{\delta L}_{}$ inductively by $\mathcal{B}_0 {:=} \mathcal{B}$ and $\mathcal{B}_{\ell +1} {:=} (\mathcal{B}_{\ell })^{\delta }_{}$. Using the corresponding single-step coarsening operators $Q_{\ell }^{} {:=} Q_{\mathcal{B}_{\ell }}^{\delta }: \mathbb {S}_{\mathrm {harm}}(\mathcal{B}_{\ell +1}) \longrightarrow \mathbb {S}_{\mathrm {harm}}(\mathcal{B}_{\ell })$ from Theorem 3.31, we make the following definition:

$$\begin{aligned} \forall u \in \mathbb {S}_{\mathrm {harm}}({\mathcal{B}}^{\delta L}_{}): \quad \quad Q_{\mathcal{B}}^{\delta ,L} u {:=} u - (\mathrm {id}- Q_{0}^{}) \circ \cdots \circ (\mathrm {id}- Q_{L-1}^{})(u) \in \mathbb {S}_{\mathrm {harm}}(\mathcal{B}). \end{aligned}$$

Using the alternative representation

$$Q_{\mathcal{B}}^{\delta ,L} u = -\sum _{\pi \in \{0,1\}^L\backslash \{0\}} (-Q_{0}^{})^{(\pi _0)} \circ \cdots \circ (-Q_{L-1}^{})^{(\pi _{L-1})} (u),$$

we infer

$$\begin{aligned} \mathrm {rank}(Q_{\mathcal{B}}^{\delta ,L})&\le \sum _{\ell =0}^{L-1} \mathrm {rank}(Q_{\ell }^{}) {\mathop {\lesssim }\limits ^{\text {Thm.~3.31}}} \sum _{\ell =0}^{L-1} ( 1 + \delta ^{-1} \mathrm {diam}_{\mathcal{T}}(\mathcal{B}_{\ell }) )^{d \sigma _{\mathrm {card}}} \\&{\mathop {\lesssim }\limits ^{\text {Lem.~3.15}}} \sum _{\ell =0}^{L-1} ( 1 + \ell + \delta ^{-1} \mathrm {diam}_{\mathcal{T}}(\mathcal{B}))^{d \sigma _{\mathrm {card}}} \le L( L + \delta ^{-1} \mathrm {diam}_{\mathcal{T}}(\mathcal{B}) )^{d\sigma _{\mathrm {card}}} \\&\le ( L + \delta ^{-1} \mathrm {diam}_{\mathcal{T}}(\mathcal{B}) )^{d\sigma _{\mathrm {card}}+1}. \end{aligned}$$

Finally, the definition of $Q_{\mathcal{B}}^{\delta ,L}$ was such that the error bound becomes elementary: For every $u \in \mathbb {S}_{\mathrm {harm}}({\mathcal{B}}^{\delta L}_{})$, iteration of Theorem 3.31 gives

$$\begin{aligned} \Vert u - Q_{\mathcal{B}}^{\delta ,L} u\Vert _{L^{2}(\mathcal{B})} = \Vert (\mathrm {id}- Q_{0}^{}) \circ \cdots \circ (\mathrm {id}- Q_{L-1}^{})(u)\Vert _{L^{2}(\mathcal{B}_0)} \le 2^{-L} \Vert u\Vert _{L^{2}({\mathcal{B}}^{\delta L}_{})}, \end{aligned}$$

which finishes the proof. $\square$

### Putting everything together

We can finally answer the question of how to find the subspace $V_{\mathcal{B},\mathcal{D},L} \subseteq L^{2}(\varOmega )$ from Sect. 3.1. After that, the Proof of Theorem 2.15 is just a matter of putting everything together.

#### Theorem 3.33

*Let* $\mathcal{T}\subseteq \mathrm {Pow}(\varOmega )$ *be a mesh of locally bounded cardinality and* $\mathcal{B}$, $\mathcal{D}\subseteq \mathcal{T}$ *clusters satisfying*

$$\begin{aligned} 0 < \mathrm {diam}_{\mathcal{T}}(\mathcal{B}) \le \sigma _{\mathrm {adm}}\mathrm {dist}_{\mathcal{T}}(\mathcal{B},\mathcal{D}). \end{aligned}$$

*Then, for every* $L \in \mathbb {N}$, *there exists a subspace*

$$\begin{aligned} V_{\mathcal{B},\mathcal{D},L} \subseteq \mathbb {S}^{p,1}_{0}(\mathcal{T}\,\,) \end{aligned}$$

*of dimension*

$$\begin{aligned} \dim {V_{\mathcal{B},\mathcal{D},L}} \lesssim L^{d\sigma _{\mathrm {card}}+1} \end{aligned}$$

*that satisfies the following approximation property: For every* $f \in L^{2}(\varOmega )$ *with* $\mathrm {supp}_{\mathcal{T}}\,(f) \subseteq \mathcal{D}$ *there holds*

$$\begin{aligned} \inf _{v \in V_{\mathcal{B},\mathcal{D},L}} \Vert S_{\mathcal{T}}\, f - v\Vert _{L^{2}(\mathcal{B})} \lesssim 2^{-L} \Vert f\Vert _{L^{2}(\mathcal{D})}. \end{aligned}$$

#### Proof

Let $\mathcal{B}$, $\mathcal{D}\subseteq \mathcal{T}$ with $0 < \mathrm {diam}_{\mathcal{T}}\,(\mathcal{B}) \le \sigma _{\mathrm {adm}}\mathrm {dist}_{\mathcal{T}}\,(\mathcal{B},\mathcal{D})$. For every given $L \in \mathbb {N}$, we make the choice $\delta {:=} \mathrm {diam}_{\mathcal{T}}\,(\mathcal{B})/(2\sigma _{\mathrm {adm}}L) > 0$ and use the space

$$\begin{aligned} V_{\mathcal{B},\mathcal{D},L} {:=}\, \mathrm {ran}(Q_{\mathcal{B}}^{\delta ,L}) \subseteq \mathbb {S}^{p,1}_{0}(\mathcal{T}\,\,). \end{aligned}$$

Here, $Q_{\mathcal{B}}^{\delta ,L}: \mathbb {S}_{\mathrm {harm}}({\mathcal{B}}^{\delta L}_{}) \longrightarrow \mathbb {S}_{\mathrm {harm}}(\mathcal{B})$ is the multi-step coarsening operator from Theorem 3.32. Using Theorem 3.32 and the definition of $\delta$, we can bound the dimension by

$$\begin{aligned} \dim {V_{\mathcal{B},\mathcal{D},L}} = \mathrm {rank}(Q_{\mathcal{B}}^{\delta ,L}) \lesssim \bigg (L + \frac{\mathrm {diam}_{\mathcal{T}}\,(\mathcal{B})}{\delta } \bigg )^{d\sigma _{\mathrm {card}}+1} \lesssim L^{d\sigma _{\mathrm {card}}+1}. \end{aligned}$$

To see the approximation properties, let $f \in L^{2}(\varOmega )$ with $\mathrm {supp}_{\mathcal{T}}\,(f) \subseteq \mathcal{D}$. By the definition of ${\mathcal{B}}^{\delta L}_{}$ and $\mathrm {dist}_{\mathcal{T}}\,({\mathcal{B}}^{\delta L}_{},\mathcal{D})$, there exist elements $B \in \mathcal{B}$, $C \in {\mathcal{B}}^{\delta L}_{}$, $D \in \mathcal{D}$ such that $\mathrm {dist}_{\mathcal{T}}\,(B,C) \le \delta L$ and $\mathrm {dist}_{\mathcal{T}}\,({\mathcal{B}}^{\delta L}_{},\mathcal{D}) = \mathrm {dist}_{\mathcal{T}}\,(C,D)$. Using the triangle inequality of the mesh metric $\mathrm {dist}_{\mathcal{T}}\,(\cdot ,\cdot )$, we conclude $\mathrm {dist}_{\mathcal{T}}\,(\mathcal{B},\mathcal{D}) \le \mathrm {dist}_{\mathcal{T}}\,(B,D) \le \mathrm {dist}_{\mathcal{T}}\,(B,C) + \mathrm {dist}_{\mathcal{T}}\,(C,D) \le \delta L + \mathrm {dist}_{\mathcal{T}}\,({\mathcal{B}}^{\delta L}_{},\mathcal{D})$. Now, exploiting the definition of $\delta$ and the assumptions on $\mathcal{B}$, $\mathcal{D}$, we obtain

$$\begin{aligned} \mathrm {dist}_{\mathcal{T}}\,({\mathcal{B}}^{\delta L}_{},\mathcal{D}) \ge \mathrm {dist}_{\mathcal{T}\,}(\mathcal{B},\mathcal{D}) - \delta L = \mathrm {dist}_{\mathcal{T}}\,(\mathcal{B},\mathcal{D}) - \frac{\mathrm {diam}_{\mathcal{T}}\,(\mathcal{B})}{2\sigma _{\mathrm {adm}}} \ge \frac{\mathrm {diam}_{\mathcal{T}}\,(\mathcal{B})}{2\sigma _{\mathrm {adm}}} > 0. \end{aligned}$$

Then, Lemma 3.26 implies $S_{\mathcal{T}}\, f \in \mathbb {S}_{\mathrm {harm}}({\mathcal{B}}^{\delta L}_{})$ and ultimately

$$\begin{aligned} \inf _{v \in V_{\mathcal{B},\mathcal{D},L}} \Vert S_{\mathcal{T}}\, f - v\Vert _{L^{2}(\mathcal{B})}&\le \Vert S_{\mathcal{T}}\, f - Q_{\mathcal{B}}^{\delta ,L}(S_{\mathcal{T}}\, f)\Vert _{L^{2}(\mathcal{B})} {\mathop {\le }\limits ^{\text {Thm.~3.31}}} 2^{-L} \Vert S_{\mathcal{T}}\, f\Vert _{L^{2}({\mathcal{B}}^{\delta L}_{})} \\&{\mathop {\lesssim }\limits ^{\text {Def.~3.15}}} 2^{-L} \Vert f\Vert _{L^{2}(\mathcal{D})}, \end{aligned}$$

which finishes the proof. $\square$

We close this section with the Proof of Theorem 2.15.

#### Proof

(of Theorem 2.15) Let $\varvec{A} \in \mathbb {R}^{N \times N}$ be the matrix from Definition 2.9 and $r \in \mathbb {N}$ a given block rank bound. We define the asserted ${\mathcal{H}}$-matrix approximant $\varvec{B} \in \mathbb {R}^{N \times N}$ to $\varvec{A}^{-1}$ in a block-wise fashion:

First, for every admissible block $(I,J) \in \mathbb {P}_{\mathrm {adm}}$, we denote the corresponding index patches by $\mathcal{B}{:=} \mathcal{T}(I) \subseteq \mathcal{T}$ and $\mathcal{D}{:=} \mathcal{T}(J) \subseteq \mathcal{T}$. From Lemma 2.12 we know that $0 < \mathrm {diam}_{\mathcal{T}}(\mathcal{B}) \le \sigma _{\mathrm {adm}}\mathrm {dist}_{\mathcal{T}}(\mathcal{B},\mathcal{D})$. Furthermore, let $C>0$ be the constant from the dimension bound in Theorem 3.33. We set $\sigma _{\mathrm {exp}}{:=} (1/C)^{1/(d\sigma _{\mathrm {card}}+1)} \ln (2) > 0$ and $L {:=} \lfloor (r/C)^{1/(d\sigma _{\mathrm {card}}+1)} \rfloor \in \mathbb {N}$. Then, Theorem 3.33 provides a subspace $V_{\mathcal{B},\mathcal{D},L} \subseteq \mathbb {S}^{p,1}_{0}(\mathcal{T}\,\,) \subseteq L^{2}(\varOmega )$. We apply Lemma 3.13 to the subspace $V_{\mathcal{B},\mathcal{D},L} \subseteq L^{2}(\varOmega )$ and get matrices $\varvec{X}_{I,J}^r \in \mathbb {R}^{I \times \tilde{r}}$ and $\varvec{Y}_{I,J}^r \in \mathbb {R}^{J \times \tilde{r}}$ of size $\tilde{r} \le \dim {V_{\mathcal{B},\mathcal{D},L}}$. We set

$$\begin{aligned} \varvec{B}|_{I \times J} {:=}\, \varvec{X}_{I,J}^r (\varvec{Y}_{I,J}^r)^T. \end{aligned}$$

Second, for every small block $(I,J) \in \mathbb {P}_{\mathrm {small}}$, we make the trivial choice

$$\begin{aligned} \varvec{B}|_{I \times J} {:=} \,\varvec{A}^{-1}|_{I \times J}. \end{aligned}$$

By Definition 2.13, we have $\varvec{B} \in {\mathcal{H}}(\mathbb {P},\tilde{r})$ with a block rank bound

$$\begin{aligned} \tilde{r} \le \dim {V_{\mathcal{B},\mathcal{D},L}} {\mathop {\le }\limits ^{\text {Thm.~3.33}}} C L^{d\sigma _{\mathrm {card}}+1} \le r. \end{aligned}$$

For the error we get

$$\begin{aligned} \Vert \varvec{A}^{-1} - \varvec{B}\Vert _{2}&{\mathop {\lesssim }\limits ^{\text {Lem.~3.12}}} \ln (N) \,\cdot \,\max _{(I,J) \in \mathbb {P}_{\mathrm {adm}}} \Vert \varvec{A}^{-1}|_{I \times J} - \varvec{X}_{I,J}^r (\varvec{Y}_{I,J}^r)^T\Vert _{2} \\ &{\mathop {\le }\limits ^{\text {Lem.~3.13}}} \ln (N) \Vert \Lambda \Vert _{}^2 \,\cdot \,\max _{\begin{subarray}{c} \mathcal{B}, \mathcal{D}\subseteq \mathcal{T}\\ \text {admissible} \end{subarray}} \sup _{\begin{subarray}{c} f \in L^{2}(\varOmega ): \\ \mathrm {supp}_{\mathcal{T}}\,(f) \subseteq \mathcal{D} \end{subarray}} \inf _{v \in V_{\mathcal{B},\mathcal{D},L}} \frac{\Vert S_{\mathcal{T}}\, f - v\Vert _{L^{2}(\mathcal{B})}}{\Vert f\Vert _{L^{2}(\mathcal{D})}} \\ &{\mathop {\lesssim }\limits ^{\text {Thm.~3.33}}} \ln (N) \Vert \Lambda \Vert _{}^2 2^{-L} \\ \lesssim & {} \ln (N) \Vert \Lambda \Vert _{}^2 \exp (-\sigma _{\mathrm {exp}}r^{1/(d\sigma _{\mathrm {card}}+ 1)}). \end{aligned}$$

Finally, it only remains to bound the norm of $\Lambda$:

$$\begin{aligned} \Vert \Lambda \Vert _{}^2 {\mathop {\lesssim }\limits ^{\text {Def.~2.6}}} h_{\min ,\mathcal{T}}^{-d} {\mathop {\lesssim }\limits ^{\text {Def.~2.4}}} h_{\mathcal{T}}^{-d\sigma _{\mathrm {card}}} {\mathop {\lesssim }\limits ^{\text {Lem.~3.1}}} \# \;\mathcal{T}\,^{\sigma_{\mathrm {card}}} \eqsim (\mathrm {dim}\,\mathbb {S}^{p,1}_{0}(\mathcal{T}\,\,))^{\sigma _{\mathrm {card}}} = N^{\sigma _{\mathrm {card}}}. \end{aligned}$$

This concludes the proof of the main result, Theorem 2.15. $\square$

## Numerical results

In this subsection, we illustrate the validity of Theorem 2.15 by means of a numerical example: For the geometry we choose the *L-shaped domain* $\varOmega {:=} ((0,1) \times (0,1)) \backslash ([1/2,1] \times [1/2,1]) \subseteq \mathbb {R}^2$ in two space dimensions. The PDE coefficients for the model problem from Sect. 2.1 are given by

$$a_1(x) {:=} \left({\begin{smallmatrix} 10 & -1 \\ -1 & 1 \end{smallmatrix}}\right),\; a_2(x) {:=} \left({\begin{smallmatrix} 10 x_2 \\ 0 \end{smallmatrix}}\right),\; a_3(x) {:=} 1.$$

The mesh $\mathcal{T}$ is *graded* in the sense of Definition 3.4 towards $\Gamma {:=} \{(1/2,1/2)\}$ with exponent $\alpha {:=} 5$ and the coarse mesh width $H {:=} 0.0095$. We use the spline space $\mathbb {S}^{1,1}_{0}(\mathcal{T}\,\,)$ ($p=1$, globally continuous, piecewise linear) and the well-known basis of *hat-functions* $\{\varphi _1,\ldots ,\varphi _N\} \subseteq \mathbb {S}^{1,1}_{0}(\mathcal{T}\,\,)$. The block partition $\mathbb {P}$ is constructed from a *geometrically balanced cluster tree* $\mathbb {T}_{N}^{}$ as suggested in [16]. We choose the parameters $\sigma _{\mathrm {adm}}{:=} 2$ and $\sigma _{\mathrm {small}}{:=} 25$ (cf. Definition 2.10). For the rank bound we choose the range $r \in \{1,\ldots ,50\}$.

Unfortunately, the ${\mathcal{H}}$-matrix approximant $\varvec{B} \in \mathbb {R}^{N \times N}$ from our proof is only a theoretical tool and inaccessible for an implementation in a computer system. Hence, we revert to a *block-wise singular value decomposition*: First, we compute the exact inverse $\varvec{A}^{-1} \in \mathbb {R}^{N \times N}$ explicitly. Then, for every admissible block $(I,J) \in \mathbb {P}_{\mathrm {adm}}$, we perform the singular value decomposition $\varvec{A}^{-1}|_{I \times J} = \varvec{U} \varvec{\Sigma } \varvec{V}^T \in \mathbb {R}^{I \times J}$. Here, $\varvec{U} \in \mathbb {R}^{I \times I}$, $\varvec{V} \in \mathbb {R}^{J \times J}$ are orthogonal and $\varvec{\Sigma } = \mathrm {diag}(\sigma _1,\ldots ,\sigma _{\min \{\# I,\# J\}}) \in \mathbb {R}^{I \times J}$ contains the corresponding singular values $\sigma _1 \ge \cdots \ge \sigma _{\min \{\# I,\# J\}} \ge 0$. Now, for the approximant we use $\varvec{B}|_{I \times J} {:=} \varvec{U}_r \varvec{\Sigma }_r \varvec{V}_r^T \in \mathbb {R}^{I \times J}$, where $\varvec{U}_r \in \mathbb {R}^{I \times r}$, $\varvec{\Sigma }_r \in \mathbb {R}^{r \times r}$ and $\varvec{V}_r \in \mathbb {R}^{J \times r}$ are the first *r* columns of $\varvec{U}$, $\varvec{\Sigma }$ and $\varvec{V}$, respectively. Recall from the theory of singular value decompositions (e.g., [21]) that

$$\begin{aligned} \Vert \varvec{A}^{-1}|_{I \times J} - \varvec{B}|_{I \times J}\Vert _{2} = \min _{\begin{subarray}{c} \varvec{C} \in \mathbb {R}^{I \times J}: \\ \mathrm {rank}(\varvec{C}) \le r \end{subarray}} \Vert \varvec{A}^{-1}|_{I \times J} - \varvec{C}\Vert _{2} = \sigma _{r+1}. \end{aligned}$$

In particular, we end up with the following *computable* error bound (cf. [21, Lemma 6.5.8])

$$\begin{aligned} \Vert \varvec{A}^{-1} - \varvec{B}\Vert _{2}&\lesssim \mathrm {depth}(\mathbb {T}_{N \times N}^{}) \,\cdot \,\max _{(I,J) \in \mathbb {P}} \Vert \varvec{A}^{-1}|_{I \times J} - \varvec{B}|_{I \times J}\Vert _{2} \\&= \mathrm {depth}(\mathbb {T}_{N \times N}^{}) \,\cdot \,\max _{(I,J) \in \mathbb {P}} \sigma _{r+1}(\varvec{A}^{-1}|_{I \times J}). \end{aligned}$$

The numerical example is implemented in Matlab. For the inversion of the full matrix $\varvec{A} \in \mathbb {R}^{N \times N}$ we use Matlab’s built-in procedure inv(...). For the singular value decompositions we use svds(...). Recall that an exact matrix inversion needs $\mathcal{O}(N^2)$ memory and $\mathcal{O}(N^3)$ time to compute, which effectively restricts the maximal feasible problem size to $N \approx 70.000$ on our machine.

**Fig. 1:**
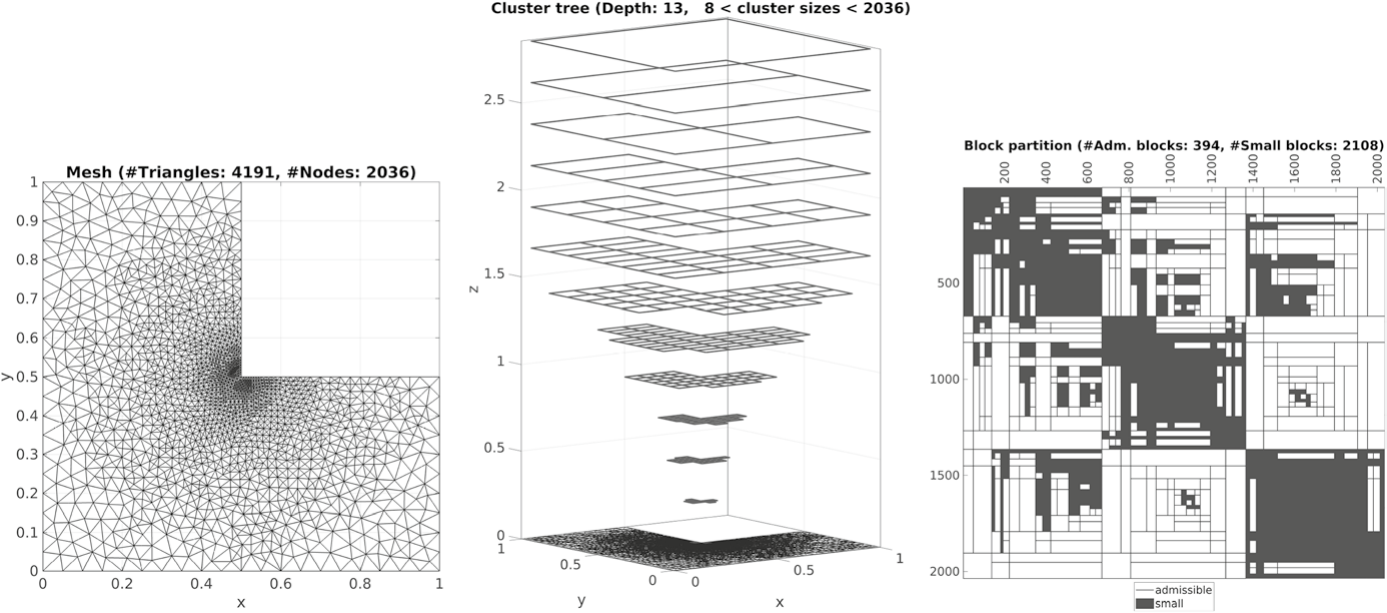
The mesh $\mathcal{T}$, the cluster tree $\mathbb {T}_{N}^{}$ and the block partition $\mathbb {P}$ for $N \approx 2.000$ degrees of freedom

In Fig. 1, we chose $N \approx 2.000$ degrees of freedom. The elements are graded towards the reentrant corner with a grading exponent $\alpha = 5$. The cluster tree $\mathbb {T}_{N}^{}$ is clearly deeper near the grading center. The block partition $\mathbb {P}$ uses sorted indices internally. Only a few admissible blocks are far away from the diagonal, lots of small blocks agglomerate along the diagonal. The sparsity pattern becomes more pronounced as $N \rightarrow \infty$.

**Fig. 2:**
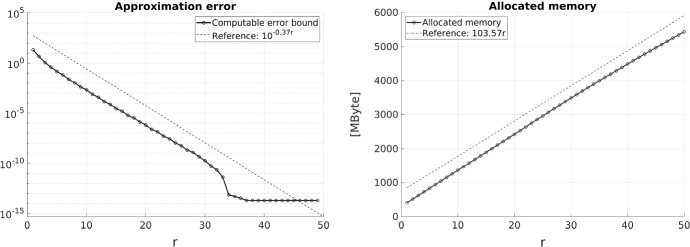
Approximation error and memory allocation for $N \approx 72.000$ degrees of freedom

In Fig. 2, we chose $N \approx 72.000$ degrees of freedom. The computable error bound from above (for $r \in \{1,\ldots ,50\}$) is depicted on a linear abscissa and a logarithmic ordinate. The values are below a straight line with slope $-0.37$ indicating an *exponential decay* $\mathrm {error}(r) \lesssim 10^{-0.37 r}$. This is even better than the asserted bound from Theorem 2.15. The allocated memory in MBytes is plotted on a linear abscissa and a linear ordinate. The values are below a straight line with slope 103.57 indicating a *polynomial growth* $\mathrm {memory}(r) \lesssim r$. Choosing a rank bound $r = 37$, for example, gives an approximation error $\approx 10^{-14}$ and uses $\approx 4.2$ GByte memory. The full system matrix takes $\approx 41.4$ GByte memory.

**Fig. 3:**
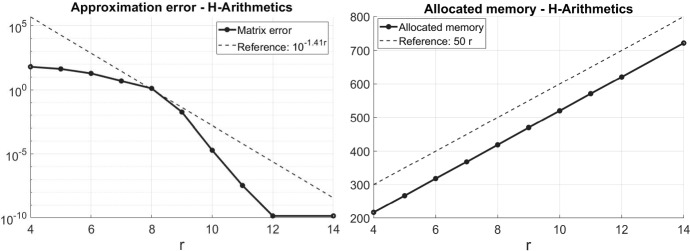
Approximation error and memory allocation (in MB) for $N \approx 30.000$ degrees of freedom using HLiB

In Fig. 3, we chose $N \approx 30.000$ degrees of freedom on a graded mesh with grading exponent $\alpha = 5$ and this time computed the ${\mathcal{H}}$-matrix approximation using the C-Library HLiB, [4]. The approximation to the inverse matrix is computed using the ${\mathcal{H}}$-matrix arithmetic of HLiB, specifically the hierarchical *LU*-decomposition. The errors shown are $\Vert \mathbf {I} - \mathbf {A}(\mathbf {L}_{{\mathcal{H}}}\mathbf {U}_{{\mathcal{H}}})^{-1}\Vert _2$, which is an upper bound for the relative error and computable without computing the inverse matrix. Again, we observe exponential convergence with respect to the rank *r* and linear growth in the memory requirements.

**Fig. 4:**
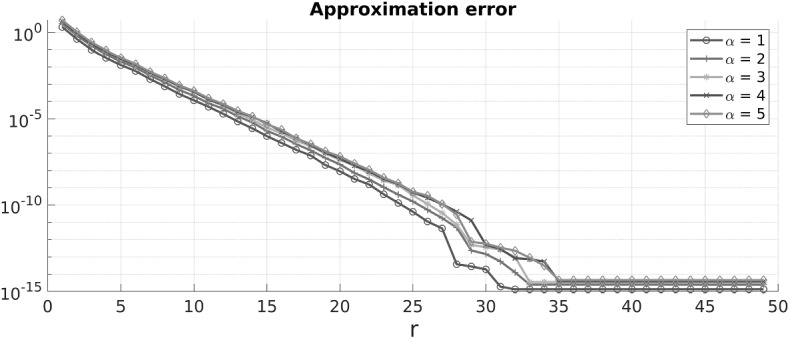
Comparison of approximation errors for different grading exponents, $\alpha \in \{1,2,3,4,5\}$. The number of degrees of freedom was kept constant at roughly $N \approx 17.500$ throughout all five runs

Finally, in Fig. 4, we chose $N \approx 17.500$ degrees of freedom and multiple grading exponents in the range $\{1,2,3,4,5\}$. The case $\alpha = 1$ corresponds to a uniform mesh, whereas $\alpha = 5$ is “heavily” graded. Again, the computable error bound from above is shown on a linear abscissa and a logarithmic ordinate. As suggested by our main result, Theorem 2.15, the convergence speed deteriorates as $\alpha$ is increased.
